# Prenatal immune activation alters the adult neural epigenome but can be partly stabilised by a n-3 polyunsaturated fatty acid diet

**DOI:** 10.1038/s41398-018-0167-x

**Published:** 2018-07-02

**Authors:** Paul Basil, Qi Li, Hongsheng Gui, Tomy C. K. Hui, Vicki H. M. Ling, Chloe C. Y. Wong, Jonathan Mill, Grainne M. McAlonan, Pak-Chung Sham

**Affiliations:** 1 Department of Psychiatry, The University of Hong Kong, Pokfulam, Hong Kong SAR China; 2 Department of Molecular & Cellular Biology, Baylor College of Medicine, Houston, TX 77030 USA; 3 State Key Laboratory of Brain and Cognitive Sciences, The University of Hong Kong, Pokfulam, Hong Kong SAR China; 4 MRC Social, Genetic and Developmental Psychiatry Centre, Institute of Psychiatry, King’s College London, De Crespigny Park, Denmark Hill, London, SE5 8AF UK; 5 University of Exeter Medical School, Exeter University, St Luke’s Campus, Magdalen Street, Exeter, EX1 2LU UK; 6 Department of Forensic and Neurodevelopmental Sciences, Institute of Psychiatry, King’s College London, De Crespigny Park, Denmark Hill, London, SE5 8AF UK; 7 Centre for Genomic Sciences, The University of Hong Kong, Pokfulam, Hong Kong SAR China

## Abstract

An unstable epigenome is implicated in the pathophysiology of neurodevelopmental disorders such as schizophrenia and autism. This is important because the epigenome is potentially modifiable. We have previously reported that adult offspring exposed to maternal immune activation (MIA) prenatally have significant global DNA hypomethylation in the hypothalamus. However, what genes had altered methylation state, their functional effects on gene expression and whether these changes can be moderated, have not been addressed. In this study, we used next-generation sequencing (NGS) for methylome profiling in a MIA rodent model of neurodevelopmental disorders. We assessed whether differentially methylated regions (DMRs) affected the chromatin state by mapping known DNase I hypersensitivity sites (DHSs), and selected overlapping genes to confirm a functional effect of MIA on gene expression using qPCR. Finally, we tested whether methylation differences elicited by MIA could be limited by post-natal dietary (omega) n-3 polyunsaturated fatty acid (PUFA) supplementation. These experiments were conducted using hypothalamic brain tissue from 12-week-old offspring of mice injected with viral analogue PolyI:C on gestation day 9 of pregnancy or saline on gestation day 9. Half of the animals from each group were fed a diet enriched with n-3 PUFA from weaning (MIA group, *n* = 12 units, *n* = 39 mice; Control group, *n* = 12 units, *n* = 38 mice). The results confirmed our previous finding that adult offspring exposed to MIA prenatally had significant global DNA hypomethylation. Furthermore, genes linked to synaptic plasticity were over-represented among differentially methylated genes following MIA. More than 80% of MIA-induced hypomethylated sites, including those affecting chromatin state and MECP2 binding, were stabilised by the n-3 PUFA intervention. MIA resulted in increased expression of two of the ‘top five’ genes identified from an integrated analysis of DMRs, DHSs and MECP2 binding sites, namely *Abat* (*t* = 2.46, *p* < 0.02) and *Gnas9* (*t* = 2.96, *p* < 0.01), although these changes were not stabilised by dietary intervention. Thus, prenatal MIA exposure impacts upon the epigenomic regulation of gene pathways linked to neurodevelopmental conditions; and many of the changes can be attenuated by a low-cost dietary intervention.

## Introduction

The epigenome exerts a modifiable control of gene expression through covalent modifications such as cytosine methylation or chromatin structural changes, which are independent of the inherent DNA sequence^1^. The epigenome is labile in nature^2^ and is thought to mediate interactions between the genome and the environment. A diverse range of environmental exposures have been associated with epigenetic modifications^3–6^; and an unstable epigenome has been linked to complex neurodevelopmental disorders, including schizophrenia^7^ and autism spectrum disorders (ASD)^8^.

Epidemiological and clinical studies indicate that prenatal exposure to maternal immune activation (MIA) is a risk factor for the subsequent development of schizophrenia^9,10^, ASD^11,12^ and bipolar disorder^13^. In rodents, this can be modelled using the viral analogue, PolyIC, to elicit an increase in inflammatory cytokine levels, especially interleukin 6 (IL6) during pregnancy^14,15^. The rodent offspring exposed to this prenatal inflammatory challenge show a robust brain and behavioural phenotype, which mirrors features of neurodevelopmental disorders^16–22^. Therefore, the consensus is that the MIA model may give a helpful insight into the mechanisms contributing to neurodevelopmental disorders in humans^23,24^. However, although postnatal inflammatory processes, and IL6 in particular, have been shown to alter the epigenome^25^ and regulate the methyltransferase gene expression^26^, little is known about the effects of a prenatal immune activation on the postnatal hypothalamic epigenome.

We previously reported the first direct experimental evidence that MIA causes global DNA hypomethylation in the hypothalamus of offspring mice^27^. This brain region is strongly implicated in the neurobiological symptoms of schizophrenia^28^ and autism^29^. In particular, there is increasing evidence that the hypothalamus is involved in the onset of ‘prodromal’ manifestations, or precursors of psychotic symptoms and structural and functional changes occur in this region during adolescence and young adulthood^30,31^. However, while this study was an important first step, we did not have access to the technology to identify MIA-induced methylation differences at the level of individual genes and gene pathways. Nor did we attempt to limit this epigenetic insult. For example, n-3 polyunsaturated fatty acid (PUFA) may have anti-inflammatory effects^32^ and a n-3 PUFA-enriched diet in individuals at ultra-high risk of developing psychiatric disorders has been reported to lead to functional improvement^33^. We reasoned that this relatively low risk intervention may be useful to examine in the MIA model.

Therefore, in this study, we used the more comprehensive approach of reduced representation bisulfite sequencing (RRBS) to quantify methylation differences at CpG dense regions of the genome^34^, with single base resolution to identify methylation differences in multiple genes. We first aimed to replicate our previous finding of global DNA hypomethylation following prenatal MIA in the mouse. We then tested the hypothesis that MIA-induced enhancer and promoter methylation is correlated with DNA hypersensitivity^35^, selective recruitment of methyl-CpG-binding protein (MECP2) at CpG dinucleotides^36^ and altered expression of downstream genes^37^. We identified biologically relevant differentially methylated regions (DMRs) and downstream target genes in an integrated analysis of DMRs, DNase I hypersensitivity sites (DHSs) and MECP2 binding sites using matching publicly available Gene Expression Omnibus (GEO) datasets. Regulation of gene expression by these epigenetic marks was tested by mRNA quantification of six transcripts from selected five ‘top-hit’ genes. Finally, we examined whether a diet enriched with n-3 PUFA introduced immediately post weaning would stabilise the MIA-affected epigenome and limit its modification.

## Materials and methods

### Sample size and experimental design

Female and male C57BL/6N mice were bred and mated in the Laboratory Animal Unit, The University of Hong Kong. Mice were held in a 12 h light–dark cycle (lights off at 1900 h), temperature and humidity-controlled (21 ± 1 °C, 55 ± 5%) animal vivarium. Animals were maintained under ad libitum food and water. All animal procedures were approved by the Committee on the Use of Live Animals in Teaching and Research at The University of Hong Kong (CULATR 2640-12) and every effort was made to minimise the number of animals used and their suffering.

Preliminary evaluations based on our pilot study^27^ suggested that ~10 individual samples give a power of 80% at an alpha = 0.05 for detecting a Δmethylation of at least 7% between the groups. The sample size in this study was calculated according to ‘experimental units’. This is considered to be an efficient and economical way to reduce variance, as all the animals exposed to the same treatment and shared the same conditions are treated as a single unit^38,39^. In this case, an experimental unit comprised same-sex litter-mates; exposed to MIA or control saline in prenatal life; fed the same diet; and housed in the same cage. In brief, of *N* = 11 pregnant mice initially used, six were exposed to PolyI:C (5 mg/kg, injection via the tail vein^22^), five to saline vehicle, on gestation day 9. We used day 9 because it is close to key developmental stages such as neural tube closure and has been reported by our group and others to reliably disrupt brain and behaviour^10,22^. This resulted in a total of *N* = 39 individual offspring exposed to MIA and *N* = 38 exposed to saline. After weaning, the same sex offspring from one litter were housed together in one experimental unit. This approach has been recommended to reduce variability within a unit to ensure any differences between conditions will be meaningful; this also increases the statistical power to detect differences^39^. Each experimental unit comprised 2–6 animals. As one of the saline-exposed mothers had delivered a large number of offspring, these were split into two units of each sex for future housing and analysis. This provided a total of 12 MIA-exposed experimental units (six male units: six female units) and 12 experimental units exposed to saline (six male units: six female units). Half the male units (three) and half the female units (three) in the MIA and saline groups were given standard diet (n-6-PUFA) and the other half were given n-3-PUFA-enriched diet. Please see Table 1 and Supplementary Figure 1 for further details.

**Table 1:** Experimental units and animal numbers

	Saline		PolyI:C	
	n-6	n-3	n-6	n-3
Units	6	6	6	6
Animals (male:female)	20 (9:11)	18 (10:8)	18 (10:8)	21 (8:13)

Saline group (*n* = 38) and polyI:C group (*n* = 39) with the number of experimental units and animals in each group

Offspring were weaned and sexed on postnatal day 21, then randomly allocated to a diet enriched with n-3 PUFA or a standard laboratory control diet (isocalorific and with the same total fat content, but a greater proportion of n-6 PUFA). The diets were custom prepared and supplied by Harlan (Harlan Laboratories UK Ltd.). The control diet, based on the standard AIN-93G rodent laboratory diet^40^, contained 65 g/kg corn oil and 5 g/kg fish oil with an approximate (n6):(n3) ratio of 13:1. The n-3 PUFA diet contained 35 g/kg corn oil and 35 g/kg menhaden fish oil with an approximate (n6):(n3) ratio of 1:1 (please see details in Supplementary Table 1). Thus, there were four groups (n-6 SAL; n-6 POL; n-3 SAL; n-3 POL) with six experimental units/group (including three males and three females). The units remained ‘naïve’—free from any subsequent interference until each animal was sacrificed by cervical dislocation at 12-weeks. The hypothalamus was rapidly dissected and frozen in liquid nitrogen until further use. DNA was isolated from brain tissue using the Qiagen EZ1 DNA kit, and equal amount of DNA each sample in the unit was combined and an equal amount of DNA was taken from each unit for analysis after quality checks and normalisation. Sample identity was concealed and uniquely coded vials were randomised at data generation and analysis stages.

### Genome-wide analysis of DNA methylation

To allow comprehensive, unbiased profiling of DNA methylation, the RRBS method was followed to capture target regions for library preparation^41^. 2.5 µg of genomic DNA was digested overnight with MspI enzyme (New England Biolabs, Ipswich, MA), using 20 units of enzyme per µg of DNA to ensure complete digestion. The size-selected targeted fragments were bisulfite converted using EZ DNA methylation kit (Zymo Research, Irvine, CA) and checked on a 2100 Bioanalyzer (Agilent Technologies, Santa Clara, CA) and Qubit 2.0 Fluorometer (Life Technologies, Grand Island, NY). A double-stranded DNA library was produced using Illumina TruSeq DNA library preparation system (Illumina, San Diego, CA). These libraries were PCR amplified, normalised and qPCR was performed to confirm quality, and were then pooled by fluorometric quantitation readings in equimolar concentrations for two lanes of a Hiseq 2000 sequencer flow cell. We performed single-end sequencing with 51 bp read length. Read quality was examined by FastQC, using Phred score of 20 as a cutoff (http://www.bioinformatics.babraham.ac.uk/projects/fastqc/). All samples were screened and deemed of high quality for inclusion in subsequent analysis.

### RRBS data processing

We aligned 16–20 million, adapter-trimmed (minimum overlap length set to 12, the minimum read length to keep after adapter removal set to 22 and allowed two mismatches), 50 nucleotides, reduced-representation bisulphite sequences from 24 experimental units. Read depths were normalised using quantile normalisation between experimental units to avoid bias introduced by systematic differences in the amount of sequences from experimental units. The quantitative methylation score at each interrogated CpG site was calculated as the ratio of the number of cytosines to the sum of the number of cytosines and thymine detected at that site. This gave a percentage of DNA methylation for each CpG site, ranging from 0 (unmethylated) to 100 (fully methylated).

### Identification of MIA and diet-associated differentially methylated regions (DMRs)

DMRs were identified in three planned comparisons. This was to address the following questions:

(i) Does MIA affect the methylome? This was addressed by comparing the MIA and saline exposure in groups given the ‘standard’ control diet (n-6-SAL vs n-6-POL).
(ii) Does dietary intervention with n-3 PUFA limit MIA-induced changes to the methylome? This was addressed by comparing the MIA group given n-3 PUFA diet and control diet (n-3-POL vs n-6-POL).
(iii) The third comparison was to address any non-specific effects of the n3-PUFA diet in the saline controls and therefore compared n-3-SAL and n-6-SAL.

### Gene or locus annotation and ranking

The DMRs identified were annotated to the nearest genomic feature using the mouse reference gene list (mm9, downloaded from the UCSC genome browser (http://genome.ucsc.edu/)). Approximately 13% of the CpG sites typed in this study spanned promoters, ~14% exons, ~21% introns and ~52% were intergenic. Similarly, ~20% of the CpG sites examined were located in CpG islands and ~5% were in shore regions. We identified ~25 000 locations in the genome with variable number of CpG sites covered. Supplementary Figure 2 shows the distances from the typed locations to the nearest feature (gene, promoter, or CpG island).

From the group comparison analysis, a number of DMRs were identified. To detect and rank biologically relevant DMRs, we considered both the statistical significance and magnitude of change of approximately 25 000 locations^8^.

1. A multiple testing false discovery rate (FDR)-based Benjamini–Hochberg correction was applied followed by filtering at a ‘*q*-value’ cut off <0.01^42^.
2. The distance from the DMR to the nearest gene was used for functional annotation.
3. The number of CpG sites covered within the same genomic region (within 250 bp) was used to identify DMRs.
4. Differentially methylated CpG sites with *q*-value < 0.01 were ranked according to the magnitude of change (Δmethylation values).

### Pathway and network analysis

DMRs with Δmethylation values more than 3% were mapped to the nearest gene^43^. Pathway and network analysis using the core analysis function from Ingenuity Systems (http://www.ingenuity.com) was used to determine what pathways incorporated these genes^44^.

### Global DNA methylation analysis

The global levels of DNA methylation at CCGG sites were evaluated using ~12 million RRBS–NGS reads by an in-house method. This method relies on the first three bases of reads beginning with CGG or TGG, which in turn depends on the methylation status of the restriction site (CCGG). Calculating the C/T ratio at this CpG site generates a measure of global CCGG methylation^45^. In effect, every read from a directional RRBS library, regardless of alignment to reference, provided information on at least one CpG at the start of the reads.

### DNase I hypersensitivity and MECP2 binding

CpG sites were annotated to the proximal mm10 enhancers from the mouse Encode project^46^. This was to identify biologically relevant sites and its affected target genes. DHSs and MECP2 occupancy sites were analysed by calculating tag density at the CpG sites using SeqMINER^47^. Default parameters were used and DHS data was filtered with a cut off tag density >5 to improve signal-to-noise ratio. Publicly available datasets of MNase sequencing (GSE66869) and MECP2 ChIP sequencing (GSE66868) were used in this analysis. These datasets were selected based on the availability of high depth DNase I hypersensitivity data and MECP2 chromatin binding data from young adult mouse hypothalamus. Effect of n-3 PUFA intervention on this list of DMRs was also tested.

### mRNA quantification

Total RNA was isolated from three biological replicates using TRIzol (Invitrogen) and cDNA was synthesised with oligo dT primers (EvoScript cDNA synthesis kit; Roche). Quantitative PCR was performed using 10 ng of cDNA and LightCycler 480 SYBR Green I reagent in a LightCycler 480 PCR apparatus (Roche). GABA transaminase (*Abat*) and *Gnas9* were chosen because they have differences in methylation in enhancer regions and *Gnas6*, spindle assembly associated (*Sfi1*), ceramide kinase (*Cerk*) and opioid receptor, kappa 1 (*Oprk1*) were chosen because they were the most statistically significant DNA methylation changes. *18S* rRNA was used as internal control. mRNA specific primer sets used in the study are listed in Supplementary Table 2. The relative standard curve method was used to get the mRNA levels in each sample and *18S* rRNA was used to normalise amounts of mRNA between samples. Statistical significance of the changes in expression levels were calculated in R project using General Linear Model (GLM).

### Statistical analysis

Statistical analyses of global DNA methylation and gene expression data used a GLM to identify any main effect of group, sex, or dietary intervention or any interaction between these factors; unpaired two-tailed *t*-tests were used to explore the data post-hoc. The criterion for statistical significance was set as *p* < 0.05 and analyses were conducted using R statistical package (http://www.r-project.org/).

MethylKit (R platform) was used to perform a principal component analysis, a cluster analysis and sample-to-sample genome-wide region-specific analysis in the planned comparisons. A logistic regression model was fitted per CpG and we tested if treatment vector has any effect on methylation or not.

$$\log \left( {\frac{{\pi _i}}{{1 - \pi _i}}} \right) = \beta _0 + \beta _1Treatment_i$$

The criterion for statistical significance was set at Benjamini–Hochberg (BH) *q* < 0.01. Ingenuity pathway analysis was used to identify pathways, networks and diseases from the top-ranked gene list employing a Fisher’s exact test for gene enrichment. The top differentially methylated sites were tested using a hypergeometrical test for over-representation in the functional annotation tools, DHS and MECP2 occupancy.

The same DMRs and genes identified as differentially methylated in the MIA-exposed brain compared to the control group were screened for any effect of n-3 PUFA using paired Wilcoxon signed rank test with continuity correction. Distribution of methylation status of these genes was tested using a Kolmogorov–Smirnov test to identify any skew in distribution.

## Results

Quantitative DNA methylation was measured across ten million CpG locations of the genome in every sample from an estimated 486 254 fragments of genomic DNA. Supplementary Figure 2 shows the distribution of percentage of methylation at all CpG locations. This example figure reveals a typical distribution from 0 to 100%, as expected from a heterogeneous sample containing a number of cell types. It also shows a peak at the 50% mark, which usually represents imprinted genes with one allele fully methylated and the other not methylated.

### Global methylation at CCGG sites are associated with MIA and n-3 PUFA intervention

Global DNA methylation in the MIA-exposed hypothalamus was significantly lower than in saline groups (*F* (3, 20) = 7.67, *β* = −5.27, *p* = 3.49 × 10^−04^) and the overall effect of n-3 PUFA dietary intervention was to increase global DNA methylation (*F* (3, 20) = 7.67, *β* = 2.534, *p* = 0.05). There was no significant effect of sex or any interaction with sex; 46.53% of the variance in global DNA methylation was explained by the regression model.

**Fig. 1:**
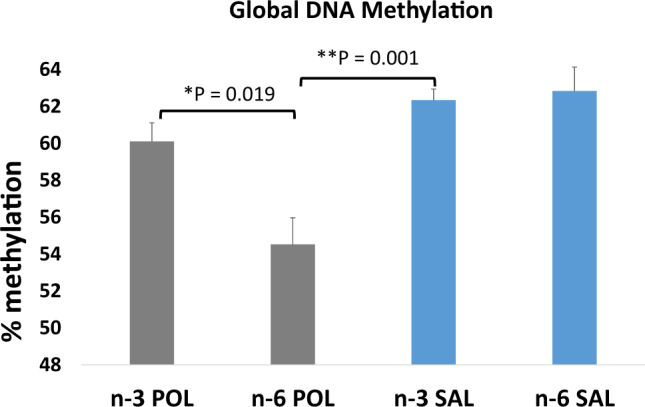
DNA hypomethylation in hypothalamus of MIA-exposed offspring. Percent methylation is shown on the *y*-axis; error bars are standard error of the mean (SEM). *p < 0.05; **p < 0.005; *p* values calculated from a stratified *t*-test between **a** maternal immune activation and control groups given a control diet and **b** MIA groups given a n-3 PUFA or control diet. Treatment groups shown on the *x*-axis: n-3 POL—Omega3 PolyI:C, n-6 POL—Omega6 PolyI:C, n-3 SAL—Omega3 Saline, and n-6 SAL—Omega6 Saline

There was a significant group × diet interaction (*F* (4, 19) = 9.96, *β* = 6.07, *p* = 0.009). This resulted from significant hypomethylation in hypothalamus of the n-6-POL compared to the n-3-POL group and was confirmed using *t*-test (*t* = 3.16, *p* = 0.011, Δmethylation = 5.57), especially in males (*t* = 4.55, *p* = 0.033, Δmethylation = 8.66). Means (SEM) are shown in Fig. 1.

### Widespread methylation differences in DNA were observed in hypothalamus

**Fig. 2:**
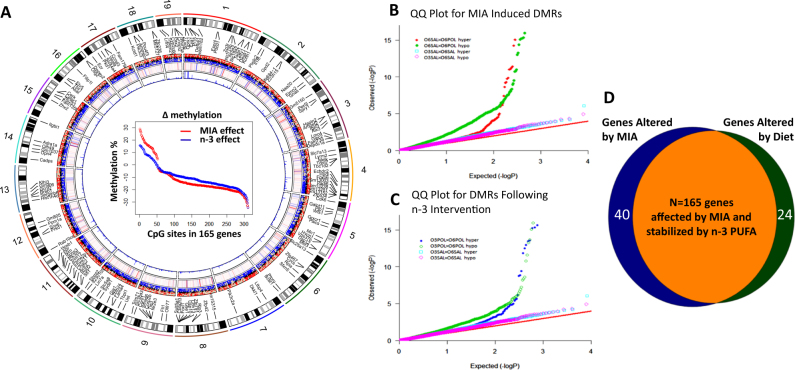
Genome-wide methylation differences in the hypothalamus. **a** MIA-induced DMRs plotted against chromosome locations, with Track1: mouse genome with cytobands. Track2: connectors between the gene names to the chromosome location. Track3: differentially methylated genes connected to the locations. Track4: red dots indicating hypermethylated sites and blue dots indicating hypomethylation. Track5: CpG site distance to the gene; red indicates after transcription start site (TSS) and blue indicates before TSS. Track6: negative log *q*-values for the corresponding CpG sites at that genomic location. Track8: comparison of Δmethylation distribution detected from MIA effect and omega-3 effect. *Y* axis indicates percentage Δmethylation values, *x* axis represents CpG sites from 165 stabilised genes. **b** Quantile–Quantile plot for MIA-induced differentially methylated CpG sites, ‘observed’ *p* values are plotted on the *y* axis against ‘expected’ values on the *x* axis. The red line indicates the distribution of *p* value under the null progression. Control group comparison is expected to follow the null and is plotted to check the level of ‘noise’. **c** Quantile–Quantile plot for differentially methylated CpG sites following dietary intervention. Observed *p* values are plotted observed on *y* axis against expected values on *x* axis. The red line indicates the distribution of *p* value under the null progression. Control group comparison is expected to follow the null and is plotted to check the level of ‘noise’. **d** Methylation profile of genes affected by MIA after n-3 intervention. (1) 165 genes stabilised by n-3 PUFA dietary intervention (Orange); (2) 40 genes affected by MIA but not affected by n-3 PUFA (Blue) and (3) not altered by MIA but altered by n-3 PUFA diet (Green)

In total, 320 differentially methylated CpG sites were identified as passing through *q*-value significance of <0.01 in a comparison between n-6-SAL vs n-6-POL. These included both hypomethylated and hypermethylated sites. Figure 2 shows the distribution of significance of differentially methylated CpG sites in MIA-exposed tissue vs saline control. The overall distribution of significance of DNA methylation differences showed a highly significant skew, with a number of CpG sites demonstrating an epigenome-wide significant difference in DNA methylation (Supplementary Table 3). A sex stratified analysis also returned similar results.

A gene annotation was performed on the DMRs and these CpG sites fell onto 204 genes, of which 36 genes have been reported to be associated with schizophrenia and four genes have been found to be associated with autism. Supplementary Figure 3 shows the genomic location of the top DMRs.

Among the genes mapped by DMRs, 164 genes were identified as hypomethylated, 33 genes were hypermethylated and seven genes had both hypomethylated and hypermethylated sites. Overall, a number of differentially methylated CpG sites passed the significance level *q*-value < 1.0 × 10^−16^ (logistic regression). The list of top differentially methylated CpG sites is given in Table 2. Nine CpG sites located in the promoter region of the gene encoding *Sfi1* were hypomethylated in the MIA-affected hypothalamus compared with control group (Δmethylation = −10.05 to 26.70%, *p* < 1.0 × 10^−16^). An enhancer region upstream to the gene *Abat* was hypomethylated in the MIA-affected hypothalamus compared with control group (Δmethylation = −14.32%, *p* < 0.004). Similarly, the promoter region of the *Gnas* transcript 9 was also hypomethylated in the MIA-affected hypothalamus compared with control group (Δmethylation = −19.74%, *p* < 4.96 × 10^−4^). Table 2 and Fig. 2 show the differentially methylated genes in this comparison.

**Table 2:** Top sites showing MIA and n-3 induced differential methylation

		**n-6-SAL vs n-6-POL**		**n-3-POL vs n-6-POL**		
**Gene name**	**Distance to the gene**	***q*** **-value**	**Δmethylation %**	***q*** **-value**	**Δmethylation %**	**CpG sites**
*Sfi1*	16	< 1.10 × 10^−16^	−26.7 to −6.59	7.18 × 10^−14^	−23.48 to −5.1	9
*Sox17*	−62863	< 1.10 × 10^−16^	10.54 to 18.23	2.58 × 10^−13^	9.58 to 16.02	6
*Pex2*	−284438	< 1.10 × 10^−16^	−17.17 to 14.54	2.60 × 10^−11^	−10.92 to 10.31	5
*Oprk1*	−237706	< 1.10 × 10^−16^	13.94 to 18.02	1.38 × 10^−10^	10.17 to 13.84	2
*Cerk*	−9086	< 1.10 × 10^−16^	−30.08 to −26.79	< 1.10 × 10^−16^	−23.16	1
*Erdr1*	−1137.5	< 1.10 × 10^−16^	−30.08 to −26.79	4.11 × 10^−13^	−27.73 to −20.44	2
*Eya1*	196687	< 1.10 × 10^−16^	16.01 to 24.08	1.30 × 10^−11^	10.50 to 18.79	2
*Filip1l*	38241	< 1.10 × 10^−16^	7.87	3.07 × 10^−13^	6.6	1
*G530011O06Rik*	−30472	< 1.10 × 10^−16^	−34.15 to −32.87	< 1.10 × 10^−16^	−31.52 to −24.74	2
*Gm15386*	−272508	< 1.10 × 10^−16^	9.02 to 13.29	1.36 × 10^−13^	8.86 to 11.66	2
*Pi15*	−134875	< 1.10 × 10^−16^	15.12	< 1.10 × 10^−16^	12.57	1
*Rn45s*	2643	< 1.10 × 10^−16^	−13.6 to 15.71	1.03 × 10^−11^	−10.53 to −7.62	3
*B020004J07Rik*	−27301	1.44 × 10^−13^	−32.83	0.001	−19.01	1
*3110070M22Rik*	−120	2.03 × 10^−13^	−9.74 to 9.93	1.03 × 10^−9^	−7.79 to 4.61	3
*Zbtb24*	−12896	1.49 × 10^−11^	−30.62	0.028	−14.75	1
*Gm10377*	82454	9.70 × 10^−10^	26.22	5.86 × 10^−4^	18.25	1
*Gm5415*	−472527	1.50 × 10^−9^	9.34	6.09 × 10^−7^	8.23	1
*4930515L03Rik*	−71170	2.40 × 10^−9^	28.79	0.166	10.06	1
*1700030C10Rik*	582097	3.59 × 10^−9^	−11.27	0.001	−7.67	1
*2900097C17Rik*	29994	4.24 × 10^−9^	−28.16	0.003	−18.63	1
*Itgbl1*	11323	3.08 × 10^−8^	26.72	0.022	15.49	1
*Plcd3*	17139	6.12 × 10^−8^	−29.14	0.004	−21.17	1
*Gm17026*	54903	1.06 × 10^−7^	23.73	0.002	17.17	1
*Rpia*	703669	1.24 × 10^−7^	22.35	1.81 × 10^−4^	17.75	1
*Mir684-1*	−41569	1.56 × 10^−7^	−26.42	0.001	−20.93	1
*9330188P03Rik*	196	2.15 × 10^−7^	−24.76	0.152	−10.96	1
*Zp3r*	28668	4.36 × 10^−7^	27.79	0.025	18.48	1
*Ppp1r15b*	40280	7.88 × 10^−7^	−27.33	0.002	−19.61	1

List of top differentially methylated genes. n-6-SAL vs n-6-POL comparison lists the MIA effect, n-3-POL vs n-6-POL comparison lists the n-3 effect. Average distance from the CpG sites to the nearest gene, *q*-value—BH corrected *p-*value, Δmethylation—difference in methylation in percentages listed as a range, number of differentially methylated CpG sites are listed in the gene. The lowest *q-*value among the differentially methylated CpG sites is listed

### Pathways and networks relevant to neurodevelopment are affected by MIA

The differentially methylated genes in n-6-SAL and n-6-POL groups that survived multiple testing correction (*q*-value < 0.01) were entered into an Ingenuity Pathway core analysis (IPA) (listed in Supplementary Table 3). IPA analysis revealed the top scoring functional networks differentially affected by MIA exposure were ‘nervous system development and function’, followed by ‘cellular development’ and ‘embryonic development’. Please see Supplementary Table 4.

### Many MIA-associated DMRs were DHSs and MECP2 affinity sites

**Fig. 3:**
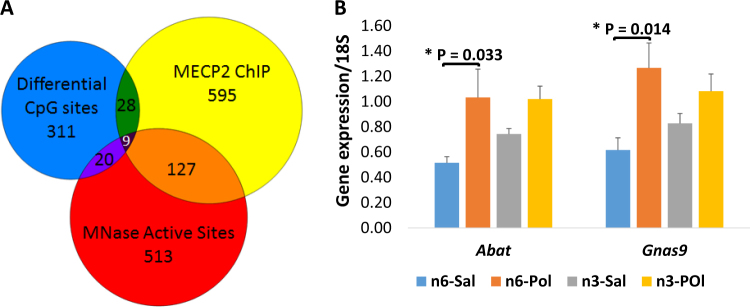
Effect of MIA-associated DMRs in gene transcription. **a** Overlap between the DMRs, DHSs and MECP2 binding sites. **b** *Abat* and *Gnas9* mRNA quantification using RT-qPCR. Quantities are calculated against a standard curve and values are normalised to 18S RNA. Error bars are standard error of the mean (SEM). *p < 0.05; *p* values calculated from a stratified *t*-test between MIA and control groups given a control diet

DHSs indicated the presence of highly expressed gene promoters, enhancers and chromatin state at the differentially methylated CpG sites. Please see Supplementary Table 5. Among the annotated DMRs, 64 sites were DHSs and MECP2 binding sites. In general, DMRs showed an overrepresentation of DHSs (hypergeometric test, *p* < 0.03) and an overrepresentation of MECP2 binding sites (hypergeometric test, *p* < 0.001). DHS or MECP2 affinity was not different at hypomethylated sites compared to hypermethylated sites. Figure 3 shows the overlap between number of DMRs, DHSs and MECP2 binding sites.

### MIA-associated differential methylation induced gene expression

Six differentially methylated sites were at annotated mouse enhancers (reference build mm10). As indicated by the bioinformatic analyses, MIA exposure-induced enhancer hypomethylation significantly increased *Abat* mRNA level in the MIA-affected group (GLM, *F* (3, 20) = 4.12, *β* = 3.96, *p* = 0.003). Please see Fig. 3. Although MIA induced hypomethylation at *Gnas6* and *Gnas9* promoter regions, only *Gnas9* mRNA was significantly increased in the MIA-affected group (*F* (3, 20) = 3.92, *β* = 4.52, *p* = 0.003). Please see Fig. 3. Finally, there was a trend towards expression changes following MIA exposure in the other selected genes.

### Dietary intervention stabilises the epigenome

In a comparison between n-3-POL and n-6-POL groups, 101 CpG sites were differentially methylated (logistic regression, *q*-value < 0.01). These included hypomethylated and hypermethylated sites. Supplementary Table 3 shows the DMRs identified in this comparison. Figure 2 shows the distribution of significance of differentially methylated CpG sites in n-3 PUFA vs control diet MIA group comparison. A comparison between n-3-SAL and n-6-SAL groups showed that the control group was not affected by n-3 PUFA administration. A sex stratified analysis also returned similar results. Differentially methylated CpG sites were annotated to the nearest gene. These significant CpG sites fell onto 64 genes of which 15 have been reported to be associated with schizophrenia and one gene (*Gnas)* was a complex imprinted locus.

Among the 64 differentially methylated genes identified, 34 were hypomethylated, 29 genes were hypermethylated and one gene had both hypo and hyper-methylated sites. The top differentially methylated sites identified were located in the promoter region of the gene encoding *Sfi1*. Eight CpG sites in this gene promoter were hypomethylated in hypothalamus of the control diet group compared with the n-3 PUFA supplemented group (Δmethylation = −8.51 to 23.48%, *p* < 1.0 × 10^−16^). The list of top differentially methylated CpG sites is given in Table 2. Figure 2 shows the number of differentially methylated genes in this comparison.

A pathway core analysis was performed on the differentially methylated genes in the n-3 PUFA MIA and control diet MIA groups that passed through multiple testing correction (*q* < 0.01); see Supplementary Table 3. IPA analysis revealed the top scoring functional network impacted by n-3 PUFA intervention was; ‘nervous system development and function’, followed by ‘cellular development’, ‘inflammatory disease’ and ‘psychological disease’. See Supplementary Table 4.

### A number of MIA-associated DMRs were stabilised by n-3 PUFA intervention

80.88% of the genes altered by MIA (165/204) were rescued by the dietary intervention. Δmethylation values for the 165 genes (affected by MIA) from n-6-SAL vs n-6-POL comparison was plotted against Δmethylation values for the corresponding genes from the n-3-POL vs n-6-POL comparison (affected by n-3 PUFA intervention). These 165 genes were identified as differentially methylated after MIA. In the MIA group with dietary intervention, this differential methylation pattern was no longer present. Thus, these genes had been stabilised by dietary intervention. Please see Fig. 2. The difference between the empirical distributions of both groups were significantly different (*D* = 0.40, Kolmogorov–Smirnov test *p* = 2.2 × 10^−16^). Furthermore, the difference between untreated comparison Δmethylation and treated comparison Δmethylation was significant (*V* = 8090, *p* < 2.2 × 10^−16^) in a paired Wilcoxon signed rank test. Pathways and functions identified from 165 genes stabilised by n-3 PUFA intervention following MIA exposure is listed in Supplementary Table 6.

### Dietary n-3 PUFA intervention alters chromatin state at MIA-associated DMRs

Among the DMRs identified as DHSs, hypomethylated sites were stabilised by dietary n-3 PUFA (*t* = 2.28, *p* = 0.02) when compared to control DMRs. Hypermethylated DHSs were not affected by dietary n-3 PUFA.

### Dietary n-3 PUFA intervention did not alter expression of selected target genes

There was no significant effect of dietary intervention, sex, any interaction with diet or sex; more than 38.23% of the variance in gene expression was explained by the regression models. Dietary n-3 PUFA intervention appeared to limit expression differences in *Sfi1, Cerk, Gnas6* in both sexes; *Gnas9* and *Oprk1* expression differences were limited by n-3 PUFA in females. However these observations did not reach statistical significance. Please see Supplementary Figure 4.

## Discussion

This study was a comprehensive analysis of DNA methylation differences in the hypothalamus from a mouse model relevant to autism and schizophrenia. It also examined the effect of n-3 PUFA early dietary intervention.

### Global DNA methylation differences and response to n-3 PUFA in offspring exposed to MIA

We documented that a global hypomethylation of CCGG locations in DNA extracted from the hypothalamus is associated with prenatal exposure to immune activation. This is consistent with the global methylation differences observed in our earlier study using a LINE1 measure^27^. The mechanism underlying this effect of MIA is unclear and may be due to both the nature of the challenge and the timing of exposure. For example, the effects of MIA are mediated at least in part by IL-6^48^ and in other systems, specifically oral cancer, IL-6 has been shown to cause global LINE-1 hypomethylation^25^. In addition, however, other prenatal exposures such as valproic acid, which are linked to adverse neurodevelopmental outcomes, have been shown to modify the epigenome including through DNA hypomethylation^49^.

The results reported here concur with evidence from human studies and other animal studies that environmental exposures have epigenetic effects^50,51^. For example, changes in global CCGG methylation in human blood samples are linked to adverse prenatal environment such as maternal exposure to famine^52^; and are also observed in the thalamus and hippocampus of mice exposed to malnourishment^53^. Moreover, the picture of predominant hypomethylation with some hypermethylation at a number of DNA sites within hypothalamic tissue of adult offspring of immune challenged mothers is broadly consistent with a recent study of prefrontal changes following MIA by Meyer group^54^.

We have now extended our previous findings and report that hypomethylation due to MIA can be limited by early dietary intervention by n-3 PUFA. These results fit with a number of studies that report beneficial effects of n-3 PUFAs on the epigenome^55^, development^56^ and in clinical treatment^57^.

### DNA methylation differences affecting chromatin state and gene expression

Extended analysis of the methylome, MNase-seq data and MECP2 ChIP-seq data showed a pattern of extensive DHS and MECP2 binding (Supplementary Figure 5) at DMRs. Mapping DHSs is an established method of identifying key transcriptional regulatory regions^58^. An overrepresentation of DHSs in the DMRs indicated there were MIA-associated differences in the transcriptional programme. The overrepresentation of MECP2 recruitment at these sites is also consistent with this.

We selected six ‘top-hits’ for mRNA analysis. First we found overexpression of *Abat* in the MIA-exposed hypothalamus. *Abat* is responsible for the catabolism of gamma-aminobutyric acid (GABA) in brain, therefore this observation fits with the evidence that GABAergic signalling is disrupted in neurodevelopmental conditions such as schizophrenia and ASD. For example, increased GABA breakdown by ABAT might contribute to the decreased GABA content reported in cerebrospinal fluid of first-episode psychosis^59^. The second gene we examined was *Gnas9. Gnas9* is a paternally expressed transcript coding the α-subunit of G-protein coupled receptors (GPCRs) such as GABA_B_ receptors. Therefore, again GABA pathways appear to be particularly targeted by MIA. This susceptibility of GABA to MIA in prefrontal cortex has previously been reported by the Meyer group^54,60,61^ and we suggest changes in methylation state are the possible antecedent. However, an alternative explanation for overexpression of *Gnas9* may be that it is a secondary response to decrease in GABA elicited by excess ABAT activity.

Finally, we observed a trend towards an increase in *Sfi1, Cerk, Gnas6* expression in MIA-exposed males and *Oprk1* expression in both sexes compared to the control group; *Sfi1, Gnas6*, and *Cerk* expression was lower in MIA-exposed females compared to the control group. Thus, it appears that at least some of the effects of MIA on gene expression are sex specific and the current stratified analysis lacked statistical power to identify any true effect.

### Specific genes, gene pathways and response to n-3 PUFA intervention

MIA-induced DNA methylation differences at individual CpG sites were concentrated within genes for ‘nervous system development and function’. Moreover, the genes modified by prenatal MIA included many of those already associated with schizophrenia and autism, including *Abat, Gnas, Cerk, Cnnm2, Pex5l, Ppp1r15b, Slc19a2, Pi15* and *Pex2*. The pathways influenced by these genes, such as the ceramide signalling pathway and AMPK signalling pathway are those involved in cell signalling and synaptic transmission fundamental to neurodevelopment and brain function^62,63^. The IL-6 signalling pathway was also enriched in this analysis of the impact of MIA. This latter observation is entirely consistent with the key role of IL6 in the MIA mouse model^15^. For example, the post-natal behavioural sequelae of prenatal MIA can be prevented by IL6 gene knock-out, or anti-IL6 antibodies^48^.

n-3 PUFA dietary supplementation from weaning attenuated the majority of MIA effects on the epigenome including genes involved in ‘regulation of transcription’; but excluding genes involved in ‘phosphoprotein phosphatase activity’. We did not examine the behavioural consequences of this intervention in the present study. This was based on increasing evidence that the post-natal environment alters neuronal metabolism and structure (e.g. Ding et al.^64^; Zhou et al.^65^) and causes epigenetic changes^66^, which might have confounded interpretation. However, we have previously shown that this same n-3 PUFA intervention prevented the pathological elevation of N-acetyl aspartate in the brains of young adult animals exposed to MIA in-utero; as well as the emergence of postnatal behavioural abnormalities^67^. This is consistent with evidence from clinical studies that n-3 PUFA supplementation may be useful in schizophrenia^68^ and in individuals at ‘ultra high risk’ for psychosis^33^.

Although the n-3 PUFA diet in general limited hypomethylation in the MIA model, and modified DHSs and MECP2 recruitment sites, it did not limit the over-expression of *Abat, Gnas9, Sfi1, Cerk, Gnas6* and *Oprk1*. This may be in part due to the range of effects of methylation on gene expression. For example, a large body of work by Bird et al. suggests that MECP2 recruitment at CpG sites within the genome is methylation density dependent and it acts as a global transcriptional repressor^69,70^. On the other hand, work by Zogbi et al. suggests that MECP2 recruitment can also activate transcription^71^. It may also be that other regulatory regions or interaction with other epimutations not influenced by n-3 PUFA contribute to the regulation of these genes and potentially explain why their expression remained unchecked. Nevertheless, inspection of the data indicated that dietary n-3 PUFA intervention did tend to limit MIA-induced changes in *Sfi1, Cerk*, and *Gnas6* expression in both sexes; and *Gnas9* and *Oprk1* in females. Possibly, n-3 PUFA effects might be specific to one sex, but this needs to be examined further in larger samples to achieve adequate statistical power.

A transcript variant-specific effect of diet was identified in *Gnas* Complex Locus (please see Supplementary Figure 6). n-3 PUFA had differential effects on two transcript variants of the *Gnas* gene including a sex-specific trend in expression; therefore its effects on overall expression may have been masked. Hypomethylation at the promoter region of transcript variant 6 was restored towards normal whereas transcript variant 9 was not significantly affected. The *Gnas* gene has been implicated in autism^72^ and other psychiatric disorders^73^, but there is currently very limited knowledge about its transcriptional complexity and how this relates to disease risk. Understanding the impact of isoform-specific differential methylation and n-3 PUFA effect on specific isoforms of *Gnas* is challenging but would be useful to explore further.

### Strengths and limitations of the study

This study had several strengths. First, an analytic approach was adopted which took account of the direction, extent and significance of methylation differences in MIA-exposed and control animals. It also allowed examination of the effect of MIA-associated differential methylation on chromatin state and gene expression. Second, a comprehensive genome-wide analysis of the role of epigenetic programming in offspring affected by environmental risk factors was performed, and the effects of genotype, age, sex were controlled for. This facilitated the discovering of DMRs in novel genomic locations as well as many of which have been previously implicated in schizophrenia and autism. Finally, it revealed a preventative effect of n-3 PUFA diet on changes in DNA methylation.

This study had a number of limitations. First, although the study had coverage of ~10 000 000 CpG sites in the mouse genome, it did not provide complete information about all promoters and CpG islands and limited by the MspI restriction sites. Moreover, validating DMRs using an alternative method if not limited by the amount of sample DNA available would have been beneficial. Second, the genome-wide methylation profiling was performed in DNA extracted from brain tissue controlling for weight, but the tissue may still be heterogeneous, and contain a range of cells of different epigenetic profile. Third, CpG sites identified as differentially methylated were annotated based on their location using the reference gene boundary, promoter location, defined CpG location and shores. This may limit identification of actual effects of CpG sites, as enhancers, chromatin modifiers and regulatory factors may act at distant sites. Fourth, chromatin state information is generated from MNase sequencing data which captures readily available open chromatin. This may generate nonspecific DNA fragments spanning a longer region of the genome. Similarly, MECP2 is an abundant neuronal protein and its high affinity for DNA may produce nonspecific ChIP enrichment. Measures were taken to reduce noise by normalising tag counts and applying a cutoff.

Finally, we did not examine the behavioural consequences of this intervention in the present study. This was based on increasing evidence that the post-natal environment also alters neuronal metabolism and structure^74,75^ and causes epigenetic changes^66^, which might have confounded interpretation. However, we have previously shown that this same n-3 PUFA intervention prevented the metabolite changes in the brains of young adult animals exposed to MIA in-utero; as well as the emergence of postnatal behavioural abnormalities^67^.

## Conclusions

In conclusion, this is the first direct examination of the impact of prenatal MIA on genome-wide DNA methylation in mouse hypothalamus. It also examined the effect of n-3 PUFA intervention on the observed methylation differences. This study shows that: (1) there was a difference in global DNA methylation between MIA-affected and saline control groups; (2) there were numerous DNA methylation differences genome-wide in the MIA-exposed adult brain; (3) many of these DMRs detected were in the promoter or key regulatory regions of novel genes and those which were implicated as risk genes in schizophrenia or autism; (4) chromatin state and expression of genes downstream to the MIA-associated DMRs was also affected; (5) a number of DMRs detected were rescued by n-3 PUFA dietary intervention.

We emphasise however, that this work has examined only one mechanism in which the environment contributes to neurodevelopmental disorders, and only one approach to modifying its detrimental impact. Our experiments were conducted under highly controlled conditions and translating our results from the rodent to the much more complex human context may not be straight-forward. They do however suggest that a fuller understanding how epigenetic mechanisms influence brain development may yield practical prevention and/or treatment targets for neurodevelopmental disorders; and the possibility of harnessing relatively ‘safe’ low-cost dietary interventions is worth further study.

### Data availability

RRBS data is available in GEO (GSE102942).

## Electronic supplementary material

Supplementary Table 1

Supplementary Table 2

Supplementary Table 3

Supplementary Table 4

Supplementary Table 5

Supplementary Table 6

Supplementary Figures Legends

Supplementary Figure 1

Supplementary Figure 2

Supplementary Figure 3

Supplementary Figure 4

Supplementary Figure 5

Supplementary Figure 6

Supplementary Figure 7
