# Supplementary material for: Prenatal immune activation alters the adult neural epigenome but can be partly stabilised by a n-3 polyunsaturated fatty acid diet
Source: Transl Psychiatry. 2018 Jul 2;8:125. doi: 10.1038/s41398-018-0167-x (PMC6028639; doi:10.1038/s41398-018-0167-x)
Supplement: Supplementary file 1 — Supplementary Table 1 [file 41398_2018_167_MOESM1_ESM.doc]

Supplementary Table 1. Table of different diet compositions used.

| **Contents (%)** | **n-3** | **n-6** | **AIN-93G** |
| --- | --- | --- | --- |
| Protein | 18.3 | 18.3 | 20 |
| Fat | 7.1 | 7.1 | 5.6 |
| LA | 1.2 | 3.72 | 2.19 |
| n-3 | 3.5 | 0.5 | 0.33 |
| n-6 | 3.5 | 6.5 | 3.78 |
| n-3:n-6 | 1:1 | 0.08:1 | 0.09:1 |

The n-6 and n-3 PUFAs were derived from corn oil or menhaden fish oil respectively. The n-6 PUFA contained 65g/Kg corn oil and 5g/Kg menhaden fish oil with an approximate (n3): (n6) ratio of 0.08:1. The n-3 PUFA diet contained 35 g/Kg corn oil and 35 g/Kg fish oil with an approximate (n3): (n6) ratio of 1:1.
