# Supplementary material for: Prenatal immune activation alters the adult neural epigenome but can be partly stabilised by a n-3 polyunsaturated fatty acid diet
Source: Transl Psychiatry. 2018 Jul 2;8:125. doi: 10.1038/s41398-018-0167-x (PMC6028639; doi:10.1038/s41398-018-0167-x)
Supplement: Supplementary file 2 — Supplementary Table 2 [file 41398_2018_167_MOESM2_ESM.docx]

**Supplementary Table 2: Primer sequences used for RT-qPCR analysis of top transcripts**.

| Gene | Primer sequence 5’- 3’ | | Length bp |
| --- | --- | --- | --- |
| *18S* | Forward | CAGGATTGACAGATTGATAGCTCTTTC | 76 |
|  | Reverse | ATCGCTCCACCAACTAAGAACG |  |
| *Abat* | Forward | CTGAACACAATCCAGAATGCAGA | 142 |
|  | Reverse | GGTTGTAACCTATGGGCACAG |  |
| *Gnas9* | Forward | GAGGACTACTTTCCAGAGTTCG | 134 |
|  | Reverse | CCACTAGCAGTGCTGATTCT |  |
| *Sfi1* | Forward | GAGGGAAGAATGGTGGGTCTC | 157 |
|  | Reverse | ATGCTCAGCTATTCGGAACCT |  |
| *Cerk* | Forward | TCCGTGCTGTGGGTGAAAC | 166 |
|  | Reverse | CGCAGTCGTCTTTTTCCTCAA |  |
| *Gnas6* | Forward | AAGCAACTGGAGGAGGAGAA | 99 |
|  | Reverse | CCGGTAACGACCCTCAGT |  |
| *Oprk1* | Forward | CGCTGTCTACTCTGTGGTATTT | 90 |
|  | Reverse | GCGGTCTTCATCTTCGTGTAT |  |

The primers were designed based on the transcribed sequence of the test gene with the PrimerQuest design software (www.idtdna.com/PrimerQuest/Home/). i). For RT-PCR the primers were designed to span for different multiple exons to avoid amplification from gDNA.
