# Supplementary material for: Prenatal immune activation alters the adult neural epigenome but can be partly stabilised by a n-3 polyunsaturated fatty acid diet
Source: Transl Psychiatry. 2018 Jul 2;8:125. doi: 10.1038/s41398-018-0167-x (PMC6028639; doi:10.1038/s41398-018-0167-x)
Supplement: Supplementary file 3 — Supplementary Table 3 [file 41398_2018_167_MOESM3_ESM.doc]

**Supplementary Table 3. Differentially methylated CpG sites n-6-SAL vs n-6-POL and n-3 POL vs n-6-POL.**

|  |  | **n-6-SAL vs n-6-POL** | | **n-3 POL vs n-6-POL** | |  |
| --- | --- | --- | --- | --- | --- | --- |
| **Gene** | **Distance to the gene** | ***q*-value** | **Δmethylation %** | ***q*-value** | **Δmethylation %** | **CpG sites** |
| *Sfi1* | 16 | < 1.10×10^-16^ | -26.70 - -6.58 | 7.18×10^-14^ | -23.48 - -5.10 | 9 |
| *Sox17* | -62850 | < 1.10×10^-16^ | -3.56 - 18.22 | 2.58×10^-13^ | -3.01 - 16.02 | 7 |
| *Pex2** | -284439 | < 1.10×10^-16^ | -17.17 - 14.53 | 2.60×10^-11^ | -10.92 - 10.31 | 5 |
| *Erdr1* | -14985 | < 1.10×10^-16^ | -30.08 - 5.57 | 4.11×10^-13^ | -27.73 - 4.42 | 3 |
| *Oprk1* | -237706 | < 1.10×10^-16^ | 13.94 - 18.01 | 1.38×10^-10^ | 10.17 - 13.84 | 2 |
| *Eya1* | 196687 | < 1.10×10^-16^ | 16.01 - 24.07 | 1.30×10^-11^ | 10.50 - 18.79 | 2 |
| *Cerk** | -9086 | < 1.10×10^-16^ | -24.4 | < 1.10×10^-16^ | -23.16 | 1 |
| *Filip1l* | 38241 | < 1.10×10^-16^ | 7.87 | 3.07×10^-13^ | 6.6 | 1 |
| *G530011O06Rik* | -30473 | < 1.10×10^-16^ | -34.15 - -32.87 | < 1.10×10^-16^ | -31.52 - -24.74 | 2 |
| *Gm15386* | -272590 | < 1.10×10^-16^ | -3.27 -13.29 | 1.36×10^-13^ | -3.08 -11.66 | 3 |
| *Pi15** | -134875 | < 1.10×10^-16^ | 15.12 | < 1.10×10^-16^ | 12.57 | 1 |
| *Rn45s* | 2549 | < 1.10×10^-16^ | -13.60 - 15.71 | 1.03×10^-11^ | -10.53 - 7.62 | 4 |
| *3110070M22Rik* | -81 | 2.03×10^-13^ | -9.74 - 9.93 | 1.03×10^-9^ | -7.78 - 4.61 | 4 |
| *B020004J07Rik* | -27307 | 1.44×10^-13^ | -32.82 - -23.05 | 1.03×10^-3^ | -19.00 - -13.94 | 2 |
| *Zbtb24* | -12917 | 1.49×10^-11^ | -30.61 - -18.08 | 0.029 | -14.74 - -6.55 | 2 |
| *Gm10377* | 175828 | 9.70×10^-10^ | 14.95 - 26.21 | 5.86×10^-4^ | 16.48 - 18.24 | 2 |
| *1700030C10Rik* | 302398 | 3.59×10^-9^ | -18.94 - -6.95 | 2.1×10^-4^ | -14.48 - -7.56 | 5 |
| *2900097C17Rik* | 29976 | 4.24×10^-9^ | -28.16 - -20.74 | 3.61×10^-3^ | -18.63 - -15.06 | 3 |
| *4930515L03Rik* | -71170 | 2.40×10^-9^ | 28.79 | 0.166 | 10.06 | 1 |
| *Gm5415* | -472527 | 1.50×10^-9^ | 9.34 | 6.09×10^-7^ | 8.23 | 1 |
| *Itgbl1* | 11323 | 3.08×10^-8^ | 26.72 | 0.022 | 15.49 | 1 |
| *Plcd3* | 17157 | 6.12×10^-8^ | -29.14 - -16.52 | 4.23×10^-3^ | -21.16 - -10.54 | 3 |
| *9330188P03Rik* | 196 | 2.15×10^-7^ | -24.76 | 0.153 | -10.96 | 1 |
| *Gm17026* | 54903 | 1.06×10^-7^ | 23.73 | 2.45×10^-3^ | 17.17 | 1 |
| *Mir684-1* | -41587 | 1.56×10^-7^ | -26.41 - -19.86 | 1.2×10^-3^ | -20.92 - -12.83 | 3 |
| *Ppp1r15b** | 40280 | 7.88×10^-7^ | -27.33 | 2.67×10^-3^ | -19.61 | 1 |
| *Rpia* | 703611 | 1.24×10^-7^ | 16.82 - 22.34 | 1.82×10^-4^ | 11.71 - 17.75 | 2 |
| *Zp3r* | 28668 | 4.36×10^-7^ | 27.79 | 0.025 | 18.48 | 1 |
| *Alkbh5* | 47487 | 4.43×10^-6^ | -26.76 - -18.96 | 0.038 | -16.10 - -13.17 | 3 |
| *Degs1* | 24844 | 8.50×10^-6^ | -21.18 - -16.88 | 0.016 | -15.42 - -9.90 | 3 |
| *Elmo2** | 5103 | 6.22×10^-6^ | -7.09 | 0.309 | -9.93 | 1 |
| *Foxo6* | 18575 | 4.61×10^-6^ | -24.01 - -12.11 | 0.096 | -11.02 - -3.99 | 6 |
| *Galnt11* | 8647 | 4.69×10^-6^ | -24.18 - -15.09 | 0.1 | -12.27 - -11.68 | 3 |
| *Gm13238* | -135 | 1.60×10^-6^ | -20.56 | 7.36×10^-3^ | -15.52 | 1 |
| *Lyrm2* | -3386 | 1.06×10^-6^ | -22.92 - -18.68 | 0.011 | -15.28 - -12.11 | 3 |
| *Pdcd1** | -98482 | 6.34×10^-6^ | 17.45 | 0.089 | 8.42 | 1 |
| *Pik3c2a* | 10535 | 5.61×10^-6^ | -21.73 | 0.021 | -15.09 | 1 |
| *5430440P10Rik* | 9587 | 4.75×10^-5^ | 22.24 | 0.576 | 5.56 | 1 |
| *Cgnl1** | -31953 | 8.79×10^-5^ | 20.42 | 0.062 | 12.74 | 1 |
| *Echdc2* | -11219 | 1.65×10^-5^ | -22.63 - -15.76 | 0.048 | -13.96 - -10.45 | 3 |
| *Gm13152* | 43645 | 2.54×10^-5^ | -10.89 | 0.03 | -7.63 | 1 |
| *Gm5150* | -45764 | 8.38×10^-5^ | 21.14 | 3.35×10^-3^ | 18.05 | 1 |
| *Gse1** | -99327 | 4.03×10^-5^ | -21.39 - -17.24 | 2.81×10^-4^ | -20.00 - -0.11 | 5 |
| *Hist1h4n* | 1467 | 4.06×10^-5^ | -21.13 - -18.66 | 0.368 | -9.35 - -7.36 | 3 |
| *Mtif3* | -30738 | 6.85×10^-5^ | -25.65 | 0.218 | -12.64 | 1 |
| *Mtif3* | -30738 | 6.85×10^-5^ | -25.65 | 0.218 | -12.64 | 1 |
| *Rn4.5s* | -3168 | 4.24×10^-5^ | -7.17 - -4.71 | 1.74×10^-5^ | -7.83 - 0.80 | 22 |
| *Slc19a2** | -24443 | 4.24×10^-5^ | -21.87 - -18.30 | 3.22×10^-4^ | -22.24 - -16.79 | 3 |
| *Usp10* | -14606 | 2.01×10^-5^ | -21.12 - -18.28 | 1.88×10^-4^ | -21.19 - -17.60 | 2 |
| *Ust* | -31629 | 1.30×10^-5^ | -21.48 | 0.11 | -11.94 | 1 |
| *Zfp251* | 17758 | 9.56×10^-5^ | 17.49 | 0.081 | 11.07 | 1 |
| *1700034H15Rik* | -26901 | 1.38×10^-4^ | -19.82 | 0.027 | -14.97 | 1 |
| *4930481A15Rik* | -275 | 2.48×10^-4^ | -17.58 - -15.03 | 0.015 | -15.35 - -13.09 | 3 |
| *4930578N18Rik* | 20597 | 6.37×10^-4^ | -18.12 | 0.167 | -11.08 | 1 |
| *4930591A17Rik* | -252129 | 5.01×10^-4^ | -22.59 | 0.517 | -8.41 | 1 |
| *Arl4c* | 33512 | 6.15×10^-4^ | -17.45 | 0.194 | -10.33 | 1 |
| *C530008M17Rik* | -25671 | 3.96×10^-4^ | -24.33 | 0.038 | -17.93 | 1 |
| *Cbfa2t3** | 11741 | 7.99×10^-4^ | -17.47 | 8.48×10^-3^ | -16.72 | 1 |
| *Ccdc185* | 16248 | 1.65×10^-4^ | -21.57 | 0.313 | -10.45 | 1 |
| *Cdk6* | -279450 | 9.5×10^-4^ | 18.93 | 0.646 | -3.55 | 1 |
| *Dopey2** | -6259 | 5.64×10^-4^ | -17.74 - -16.00 | 0.102 | -11.92 - -9.41 | 2 |
| *Emx2* | 2729 | 5.86×10^-4^ | -18.45 | 0.054 | -12.92 | 1 |
| *Ezr** | 33548 | 2.36×10^-4^ | -20 | 2.45×10^-3^ | -19.77 | 1 |
| *F830002L21Rik* | 28745 | 5.86×10^-4^ | -18.19 | 0.151 | -10.8 | 1 |
| *Fam179a* | 1911 | 5.53×10^-4^ | -20.93 | 0.169 | -12.38 | 1 |
| *Gcnt2* | 9477 | 6.08×10^-4^ | -20.73 | 0.543 | -8.11 | 1 |
| *Gm805* | -10474 | 1.14×10^-4^ | -21.48 | 0.096 | -14.08 | 1 |
| *Gnas** | 1880 | 4.96×10^-4^ | -19.74 - -15.01 | 0.138 | -11.37 - -6.01 | 3 |
| *Hpdl* | -18626 | 2.94×10^-4^ | -22.03 | 0.211 | -11.53 | 1 |
| *Hus1* | 90026 | 1.75×10^-4^ | -19.01 | 0.015 | -15.69 | 1 |
| *L3mbtl1* | 4726 | 4.60×10^-4^ | -18.78 - -17.14 | 0.778 | 1.20 - 2.81 | 2 |
| *Ltbp4** | -990 | 9.89×10^-4^ | -18.43 | 0.679 | -3.99 | 1 |
| *Mir5623* | 115078 | 9.02×10^-4^ | -20.23 | 0.146 | -13.07 | 1 |
| *Naa20* | -42157 | 9.93×10^-4^ | -18.49 | 0.734 | -3.65 | 1 |
| *Olfr49* | -14315 | 1.57×10^-4^ | -18.79 - -16.70 | 0.015 | -14.81 - -13.42 | 3 |
| *Opn4* | -14648 | 7.29×10^-4^ | -22.85 | 0.608 | -6.74 | 1 |
| *Padi6* | -8072 | 4.60×10^-4^ | -22.33 | 0.116 | -13.81 | 1 |
| *Paics** | -20107 | 4.60×10^-4^ | -18.37 - -17.38 | 0.011 | -16.07 - -13.41 | 2 |
| *Plxdc2* | -327618 | 5.86×10^-4^ | -19.23 - -18.13 | 0.047 | -14.46 - -11.30 | 2 |
| *Ppm1a* | -11032 | 2.71×10^-4^ | -20.46 | 0.173 | -12.18 | 1 |
| *Rab10os* | -167454 | 1.25×10^-4^ | 20.05 | 0.174 | 11.73 | 1 |
| *Slc35g1* | -20171 | 2.19×10^-4^ | -19.42 - -19.39 | 0.173 | -11.09 - -10.79 | 2 |
| *Slc44a4* | -1706 | 6.08×10^-4^ | -17.92 | 0.751 | -3.43 | 1 |
| *Smad6* | 23831 | 7.74×10^-4^ | -17.76 | 0.024 | -15.1 | 1 |
| *Spns3* | -26298 | 9.93×10^-4^ | -12.62 | 0.256 | -6.45 | 1 |
| *Tbc1d2* | -169473 | 2.71×10^-4^ | -20.98 | 0.435 | -9.31 | 1 |
| *Tbpl1* | -3670 | 2.83×10^-4^ | -21.44 - -16.12 | 5.58×10^-4^ | -22.09 - -13.13 | 2 |
| *Trim71* | 16105 | 7.36×10^-4^ | -21.48 | 0.294 | -11.1 | 1 |
| *Zfp236* | -1632 | 3.43×10^-4^ | -18.6 | 0.152 | -11.04 | 1 |
| *Zfp42* | -12792 | 2.19×10^-4^ | -20.44 - -20.07 | 0.24 | -10.64 - -8.09 | 2 |
| *Zfp791* | -8468 | 3.37×10^-4^ | -18.93 | 0.011 | -16.78 | 1 |
| *Zfp954* | 43395 | 6.67×10^-4^ | -17.53 - -17.44 | 0.017 | -15.07 - -13.02 | 2 |
| *Zhx2* | 38762 | 1.64×10^-4^ | -19.39 | 0.151 | -10.43 | 1 |
| *1700007B14Rik* | 264444 | 4.14×10^-3^ | -18.24 | 0.52 | -8.14 | 1 |
| *1700024P04Rik* | -9918 | 7.35×10^-3^ | -16.4 | 0.169 | -11.87 | 1 |
| *2210420H20Rik* | -5472 | 7.54×10^-3^ | -16.53 | 0.236 | -10.98 | 1 |
| *2410018L13Rik* | 735207 | 2.23×10^-3^ | -21.32 | 0.111 | -14.26 | 1 |
| *2610035D17Rik* | 170178 | 6.99×10^-3^ | -14.31 | 1.65×10^-3^ | -17.61 | 1 |
| *4930455B14Rik* | 133976 | 1.02×10^-3^ | -19.37 | 0.057 | -13.98 | 1 |
| *4930543E12Rik* | -15116 | 1.21×10^-3^ | -19.29 | 0.474 | -7.28 | 1 |
| *4930554C24Rik* | -115476 | 3.58×10^-3^ | 11.72 - 13.22 | 0.434 | 4.52 - 6.03 | 2 |
| *5730507C01Rik* | 729473 | 3.36×10^-3^ | -15.84 - -15.08 | 0.129 | -12.83 - -11.21 | 2 |
| *9230105E05Rik* | 43279 | 3.76×10^-3^ | -16.35 | 0.386 | -7.67 | 1 |
| *Abat*^#^* | 16717 | 4.25×10^-3^ | -14.32 | 0.701 | -3.52 | 1 |
| *Abr** | 21719 | 6×10^-3^ | -16.34 | 0.176 | -11.31 | 1 |
| *Adgrd1* | 22300 | 7.9×10^-3^ | -16.13 | 0.119 | -12.78 | 1 |
| *Adra1a* | -42925 | 6.47×10^-3^ | -13.32 | 0.776 | -2.82 | 1 |
| *Ak4* | 257 | 7.2×10^-3^ | -14.29 | 0.494 | -5.95 | 1 |
| *Alx3* | 5607 | 5.3×10^-3^ | -8.49 | 0.876 | -0.78 | 1 |
| *Ammecr1l* | 347 | 8.76×10^-3^ | -11.53 | 0.377 | -5.72 | 1 |
| *C130060K24Rik* | 26350 | 8.47×10^-3^ | -9.41 | 0.434 | -4.9 | 1 |
| *Ccnd3* | 40869 | 7.02×10^-3^ | -15.63 | 0.384 | -8.42 | 1 |
| *Cdh13** | -94335 | 6.87×10^-3^ | -15.79 | 0.328 | -9.28 | 1 |
| *Celsr1** | 55110 | 7.13×10^-3^ | 4.58 | 0.108 | 3.06 | 1 |
| *Cep85* | 27050 | 2.31×10^-3^ | -15.94 - -14.38 | 2.23×10^-3^ | -17.75 - -16.93 | 2 |
| *Chac2* | -49448 | 2.99×10^-3^ | 10.1 | 0.054 | 8.58 | 1 |
| *Cnnm2** | 32726 | 6.99×10^-3^ | -19.4 | 0.452 | -8.8 | 1 |
| *Coro2b** | 97208 | 2.03×10^-3^ | -17.58 - -17.56 | 0.031 | -15.94 - -14.96 | 2 |
| *Cycs** | 7 | 3.58×10^-3^ | -20.84 - -20.51 | 0.525 | -7.76 - -7.42 | 2 |
| *Cyp2c66* | 43452 | 1.08×10^-3^ | -13.23 | 0.173 | -7.98 | 1 |
| *D730045A05Rik* | -47162 | 9.43×10^-3^ | -15.27 | 0.482 | -7.51 | 1 |
| *Depdc1a* | 94 | 1.37×10^-3^ | -11.17 | 0.1 | -7.81 | 1 |
| *Enpp7* | -31044 | 6.87×10^-3^ | -14.94 | 0.243 | -8.8 | 1 |
| *Espnl* | -11 | 1.18×10^-3^ | -18.01 | 0.057 | -15.07 | 1 |
| *Etv5* | 9202 | 8.47×10^-3^ | -14.14 | 0.338 | -8.11 | 1 |
| *Fam135b** | 794590 | 3.03×10^-3^ | -13.55 | 0.116 | -10.38 | 1 |
| *Fam196b* | 101785 | 8.21×10^-3^ | -14.25 | 0.503 | -6.23 | 1 |
| *Fam73a* | 16437 | 3.25×10^-3^ | -16.36 - -15.64 | 0.338 | -8.71 --8.68 | 2 |
| *Fbxw11* | 540 | 2.08×10^-3^ | -13.71 | 0.683 | -3.2 | 1 |
| *Fli1* | -2460 | 3.55×10^-3^ | -16.38 | 0.055 | -13.67 | 1 |
| *Foxl1* | 146260 | 6.56×10^-3^ | -16.26 | 0.2 | -10.77 | 1 |
| *Gal3st2* | -16060 | 2.99×10^-3^ | 9.19 | 0.042 | 7.76 | 1 |
| *Gipc2* | 72936 | 3.16×10^-3^ | -17.07 | 0.073 | -13.07 | 1 |
| *Gja1** | 330663 | 6.19×10^-3^ | -3.73 | 0.916 | 0.05 | 1 |
| *Gm10578* | -24847 | 7.44×10^-3^ | -12.28 | 0.575 | -5.71 | 1 |
| *Gm13003* | 36720 | 2.91×10^-3^ | -18.47 | 0.047 | -15.83 | 1 |
| *Gm15315* | 13690 | 3.71×10^-3^ | 15.71 | 0.27 | 8.47 | 1 |
| *Gm20063* | 367266 | 8.78×10^-3^ | 8.44 | 0.119 | 6.69 | 1 |
| *Gm20871* | 113304 | 6.18×10^-3^ | -16.44 - -15.96 | 4.18×10^-3^ | -19.02 - -17.47 | 2 |
| *Gm906* | 228769 | 2.75×10^-3^ | 8.87 | 0.494 | 3.16 | 1 |
| *Grk5* | -473 | 1.68×10^-3^ | -17.14 | 0.21 | -9.94 | 1 |
| *H2afy3* | -346820 | 3.85×10^-3^ | -12.56 | 0.011 | -12.52 | 1 |
| *Hapln2** | 3809 | 5.3×10^-3^ | -12.17 | 0.668 | -3.43 | 1 |
| *Hoxb1^#^* | 41894 | 2.48×10^-3^ | -11.89 | 0.677 | -2.94 | 1 |
| *Hvcn1* | -16024 | 8.32×10^-3^ | -16.73 | 0.2 | -12.12 | 1 |
| *Igsf3* | 27741 | 4.31×10^-3^ | -14.66 | 0.04 | -13.15 | 1 |
| *Il1f8* | 14800 | 7.54×10^-3^ | 6.78 | 0.075 | 5.7 | 1 |
| *Kcnh1** | 253649 | 9.59×10^-3^ | -15.73 | 0.117 | -13.01 | 1 |
| *Kctd1* | 425 | 5.63×10^-3^ | -11.01 | 0.286 | -6.25 | 1 |
| *Klhl3* | 27787 | 1.42×10^-3^ | -19.15 | 0.05 | -14.94 | 1 |
| *Map1b* | -126167 | 6.68×10^-3^ | -15.69 | 0.288 | -10.29 | 1 |
| *Melk* | 172793 | 1.96×10^-3^ | 16.96 | 0.133 | 11.44 | 1 |
| *Mif4gd* | 3955 | 3.46×10^-3^ | -19.29 | 0.237 | -11.76 | 1 |
| *Mir139* | -9777 | 1.93×10^-3^ | -19.21 | 0.592 | -5.93 | 1 |
| *Mir5127* | 93399 | 1.2×10^-3^ | -18.43 | 0.893 | 1.24 | 1 |
| *Mir7226* | -15284 | 3.81×10^-3^ | 14.16 | 4.2×10^-3^ | 15.55 | 1 |
| *Mn1* | 2780 | 1.37×10^-3^ | -21.12 | 0.091 | -16.02 | 1 |
| *Nr2e3** | 2673 | 6.12×10^-3^ | -17.16 | 2.33×10^-3^ | -21.33 | 1 |
| *Nr5a2* | 3905 | 7.03×10^-3^ | -11.72 | 0.72 | -2.8 | 1 |
| *Nxn* | 16454 | 1.5×10^-3^ | -17.49 | 0.224 | -11.25 | 1 |
| *Olfr77* | -11391 | 3.37×10^-3^ | -14.32 - -13.71 | 0.016 | -15.01 - -14.21 | 2 |
| *Olig2* | -48170 | 4.16×10^-3^ | 13.96 | 0.1 | 10.89 | 1 |
| *Pax2* | -58360 | 8.87×10^-3^ | -12.28 | 0.892 | 0.67 | 1 |
| *Pik3c3* | -5611 | 2.61×10^-3^ | -16.27 | 0.018 | -15.66 | 1 |
| *Pik3r5* | 118 | 6.99×10^-3^ | -14.88 | 0.631 | -4.71 | 1 |
| *Plb1* | -30884 | 5.71×10^-3^ | -15.42 | 0.039 | -14.86 | 1 |
| *Pold2* | -8990 | 5.24×10^-3^ | -16.59 | 0.297 | -9.92 | 1 |
| *Pou4f3* | 22828 | 6×10^-3^ | -13.92 | 0.426 | -6.54 | 1 |
| *Ppp1r2-ps9* | -363374 | 7.76×10^-3^ | -14.41 | 0.399 | -7.89 | 1 |
| *Psme2b* | -5434 | 4.98×10^-3^ | 16.46 | 0.026 | 16.19 | 1 |
| *Ptpro* | 43156 | 7.49×10^-3^ | -17.22 | 1.09×10^-4^ | -25.46 | 1 |
| *Rai1*^#^* | -30880 | 3.03×10^-3^ | -16.23 | 0.626 | -5.06 | 1 |
| *Rims1** | -127746 | 5.71×10^-3^ | -11.38 | 0.502 | -5.45 | 1 |
| *Rln3* | 142 | 2.43×10^-3^ | -18.12 | 0.39 | -8.15 | 1 |
| *Rreb1** | 20423 | 8.1×10^-3^ | -15.26 | 0.449 | -7.83 | 1 |
| *Serpinb6e* | 66484 | 1.64×10^-3^ | 20.37 | 0.531 | 2.72 | 1 |
| *Sfxn5** | 45380 | 8.53×10^-3^ | -15.94 | 0.213 | -11.34 | 1 |
| *Shmt2** | -4843 | 3.59×10^-3^ | -13.36 | 0.084 | -10.79 | 1 |
| *Shtn1* | 18182 | 6.14×10^-3^ | -17.82 | 0.724 | -4.98 | 1 |
| *Slc13a2os* | 3085 | 4.16×10^-3^ | -12.89 | 0.022 | -12.18 | 1 |
| *Slc25a13** | 17466 | 6.72×10^-3^ | -12.28 | 0.173 | -8.44 | 1 |
| *Slc5a8* | 160152 | 6.19×10^-3^ | 13.72 | 0.396 | 6.87 | 1 |
| *Smim12* | -28944 | 6.54×10^-3^ | -15.47 | 0.212 | -10.75 | 1 |
| *Snx6* | 20645 | 2.03×10^-3^ | -16.66 - -16.28 | 4.87×10^-3^ | -16.72 - -12.61 | 2 |
| *Spry1* | 231 | 3.03×10^-3^ | -16.99 | 0.364 | -8.2 | 1 |
| *Sult5a1* | -7268 | 3.77×10^-3^ | -16.70 - -15.36 | 0.585 | -5.72 - -2.62 | 2 |
| *Syde1* | 934 | 1.4×10^-3^ | -16.93 | 0.103 | -11.67 | 1 |
| *Tbx1*^#^* | 855 | 3.39×10^-3^ | -16.88 | 0.139 | -11.79 | 1 |
| *Tdrd7* | -21571 | 1.57×10^-3^ | -16.74 | 0.544 | -5.88 | 1 |
| *Tle3** | 137419 | 1.57×10^-3^ | -18.36 | 0.483 | -6.87 | 1 |
| *Tmem216** | 14998 | 1.86×10^-3^ | 17.18 | 0.173 | 10.93 | 1 |
| *Tpgs1* | 6247 | 2.08×10^-3^ | -16.62 | 0.516 | -6.1 | 1 |
| *Usp12* | 81949 | 6.41×10^-3^ | -17.15 | 0.088 | -14.12 | 1 |
| *Vegfa* | -2613 | 2.17×10^-3^ | -9.64 | 0.078 | -7.12 | 1 |
| *Vps37b* | 6289 | 6.2×10^-3^ | -16.28 | 0.355 | -8.96 | 1 |
| *Wfs1* | 52535 | 6.47×10^-3^ | -15.85 | 0.104 | -12.16 | 1 |
| *Zfp467* | 6680 | 6.57×10^-3^ | -12.73 | 0.869 | -1.33 | 1 |
|  |  |  |  |  |  |  |

n-6-SAL vs n-6-POL comparison lists the MIA effect. n-3 POL vs n-6-POL comparison lists the n-3 effect. Distance to the nearest gene, *q*-value –BH corrected *p-*value, **Δ**methylation – difference in methylation in percentages listed as a range, number of differentially methylated CpG sites are listed in the gene. The lowest *q*-value among the differentially methylated CpG sites is listed for these genes. * Functional association with Schizophrenia. # Association with autism.
