# Supplementary material for: Prenatal immune activation alters the adult neural epigenome but can be partly stabilised by a n-3 polyunsaturated fatty acid diet
Source: Transl Psychiatry. 2018 Jul 2;8:125. doi: 10.1038/s41398-018-0167-x (PMC6028639; doi:10.1038/s41398-018-0167-x)
Supplement: Supplementary file 4 — Supplementary Table 4 [file 41398_2018_167_MOESM4_ESM.doc]

**Supplementary Table 4**. Ingenuity Pathway Analysis of the MIA affected genes.

|  | **Functional network** | **Biofunction Pathway** | **Canonical Pathway** | **Physiological System Developmentand Function** |
| --- | --- | --- | --- | --- |
| 1) n-6-SAL vs n-6-POL | Nervous system development and function (34) | Developmental disorders(22,  *p* = 5.69×10^-5^, *q* = 0.01) | Ceramide signalling  (*p* = 3.53×10^-4^),  AMPK Signalling  ( *p* = 5.72×10^-4^),  IL-6 signalling  (*p* = 1.76×10^-3^) | Connective Tissue Development and Function (16, *p* = 2.38×10^-6^, *q* = 0.01),  Embryonic Development  (37, *p* = 2.38×10^-6^, *q* = 0.01),  Organ Development (26,  *p* = 2.38×10^-6^, *q* = 0.01) |
| 2) n-3-POL vs n-6-POL | Nervous system development and function (27) | Developmental disorders, 4th (5, *p* = 3.24×10^-3^,  *q* = 0.04) | Neuropathic pain signalling in dorsal horn neurons  (*p* = 3.09×10^-4^) | Nervous system development and function (11, *p* = 2.5×10^-3^, *q* = 0.04),  Embryonic Development  (15, *p* = 3.24×10^-3^, *q* = 0.04) |

*q*-value – BH corrected *p-*value, 1) The top biological function pathway in this comparison was ‘developmental disorders’ comprising 22 differentially methylated genes. 2) Among the top biological functions, ‘developmental disorders’ comprising 5 differentially methylated genes was ranked four. The top canonical pathway identified was ‘neuropathic pain signalling in dorsal horn neurons’ and the next ranked pathways were signalling pathways. The top ranking functions within molecular and cellular functions were carbohydrate and lipid metabolism related.
