# Supplementary material for: Prenatal immune activation alters the adult neural epigenome but can be partly stabilised by a n-3 polyunsaturated fatty acid diet
Source: Transl Psychiatry. 2018 Jul 2;8:125. doi: 10.1038/s41398-018-0167-x (PMC6028639; doi:10.1038/s41398-018-0167-x)
Supplement: Supplementary file 5 — Supplementary Table 5 [file 41398_2018_167_MOESM5_ESM.docx]

**Supplementary Table 5. Chromatin state and MECP2 occupancy at DMRs**

| **Gene** | **MNase Tags** | **MECP2 ChIP Tags** | **Distance to Nearest Feature** |
| --- | --- | --- | --- |
| Sfi1 | 24 | 1 | -94763 |
| Rn45s | 28 | 2 | -94740 |
| Trim71 | 22 | 2 | -91317 |
| Mif4gd | 28 | 3 | -48170 |
| Naa20 | 2 | 5 | -42157 |
| Vps37b | 10 | 2 | -31953 |
| Slc13a2os | 20 | 2 | -31044 |
| Klhl3 | 16 | 2 | -30884 |
| Smad6 | 4 | 2 | -30880 |
| Rn45s | 3 | 1 | -30738 |
| Ppp1r15b | 18 | 2 | -28944 |
| Fam179a | 5 | 3 | -26298 |
| Gcnt2 | 8 | 3 | -24847 |
| Zhx2 | 17 | 3 | -21571 |
| L3mbtl1 | 31 | 3 | -15284 |
| Cbfa2t3 | 3 | 2 | -11394 |
| L3mbtl1 | 56 | 1 | -10474 |
| Gipc2 | 20 | 1 | -9086 |
| Tdrd7 | 21 | 1 | -8990 |
| Sfxn5 | 22 | 2 | -2460 |
| Rreb1 | 5 | 2 | -1706 |
| Spns3 | 18 | 2 | -990 |
| Gm10578 | 13 | 2 | -473 |
| Olig2 | 31 | 2 | -11 |
| Mir7226 | 573 | 10 | 36 |
| Gse1 | 2 | 1 | 94 |
| Ltbp4 | 16 | 2 | 540 |
| Espnl | 175 | 1 | 1854 |
| Slc44a4 | 71 | 3 | 1911 |
| Nxn | 18 | 2 | 2041 |
| Gse1 | 31 | 1 | 2673 |
| Ptpro | 143 | 4 | 3032 |
| Grk5 | 261 | 6 | 3044 |
| Tpgs1 | 23 | 5 | 3085 |
| Fli1 | 11 | 1 | 3905 |
| Rai1 | 60 | 5 | 3955 |
| Smim12 | 55 | 3 | 4712 |
| Plb1 | 78 | 3 | 4740 |
| Gnas | 50 | 2 | 6247 |
| Enpp7 | 29 | 5 | 6289 |
| Fbxw11 | 24 | 2 | 6680 |
| Olfr77 | 9 | 1 | 9202 |
| Zfp467 | 6 | 3 | 9477 |
| Tmem216 | 14 | 1 | 11323 |
| Cgnl1 | 8 | 3 | 11741 |
| Mtif3 | 13 | 2 | 14998 |
| Cerk | 30 | 5 | 16105 |
| Foxo6 | 1 | 2 | 16454 |
| Gm805 | 13 | 1 | 16717 |
| Gse1 | 50 | 1 | 17758 |
| Ezr | 18 | 1 | 18207 |
| Gm13003 | 12 | 3 | 20423 |
| Nr2e3 | 28 | 4 | 23831 |
| Pold2 | 1 | 4 | 27787 |
| Wfs1 | 3 | 1 | 33548 |
| Ccnd3 | 13 | 1 | 36720 |
| Abat | 2 | 3 | 38762 |
| Etv5 | 13 | 3 | 40280 |
| Rn45s | 10 | 1 | 40869 |
| Hoxb1 | 6 | 1 | 41894 |
| Nr5a2 | 1 | 2 | 43156 |
| Depdc1a | 6 | 3 | 45380 |
| Zfp251 | 8 | 1 | 52535 |
| Itgbl1 | 11 | 3 | 72936 |

MNase, MECP2 ChIP tag intensity at the DMRs and distance to the nearest gene is listed.
