# Supplementary material for: Prenatal immune activation alters the adult neural epigenome but can be partly stabilised by a n-3 polyunsaturated fatty acid diet
Source: Transl Psychiatry. 2018 Jul 2;8:125. doi: 10.1038/s41398-018-0167-x (PMC6028639; doi:10.1038/s41398-018-0167-x)
Supplement: Supplementary file 6 — Supplementary Table 6 [file 41398_2018_167_MOESM6_ESM.doc]

**Supplementary Table 6**. Ingenuity Pathway Analysis of the n-3 affected and unaffected genes intervention.

|  | **Brain expression** | **Biofunction Pathway** |
| --- | --- | --- |
| Intervention affected | 65 genes, *p* = 1.9×10^-3^, *q* = 0.16 | Regulation of transcription, DNA-dependent (24 genes, *p* = 2.01×10^-4^) |
| Intervention unaffected | 14 genes, *p* = 0.043, *q* = 0.91 | Phosphoprotein phosphatase activity (3 genes, *p* = 0.02) |
| Intervention specific DMRs | Not specific | Vasopressin-regulated water reabsorption, (*p* = 4×10^-3^), Proton Pump Inhibitor Pathway (*p* = 4.1×10^-3^). |

*q-*value – BH corrected *p-*value, 1) Brain was the active site of expression for 65 genes and the top biological function pathways affected were ‘regulation of transcription, DNA dependent’. No significant canonical pathways were identified. 2) Similarly, pathway and functional analysis of 40 genes not affected by n-3 intervention showed 14 genes actively expressed in brain. The top biological function identified in this subset of genes was ‘phosphorprotein phosphatase activity’.
