# Supplementary material for: Prenatal immune activation alters the adult neural epigenome but can be partly stabilised by a n-3 polyunsaturated fatty acid diet
Source: Transl Psychiatry. 2018 Jul 2;8:125. doi: 10.1038/s41398-018-0167-x (PMC6028639; doi:10.1038/s41398-018-0167-x)
Supplement: Supplementary file 7 — Supplementary Figures Legends [file 41398_2018_167_MOESM7_ESM.docx]

**Supplementary Figure 1. The assignment of offspring and litters to the experimental groups ("units").**

**Supplementary Figure 2. Analytical pipeline used in the study.**

**Supplementary Figure 3. Quality control and annotations.**

**A.** Distribution of DMRs annotated to genomic locations. **B.** Distribution of DMRs to CpG islands and shores. **C.** Frequency distribution showing distance from DMRs to the nearest feature. **D.** Distribution of methylation percentages at the investigated CpG sites.

**Supplementary Figure 4. Genome Browser tracks for the top differentially methylated genes.**

Y axis shows percentage of methylation for saline control group and MIA group tracks. **A.** Methylation profile of CpG sites within *Sfi1.* **B.** Methylation profile of CpG sites within *Sox17.* **C.** Methylation profile of CpG sites within *Pex2*.

**Supplementary Figure 5. Gender stratified gene expression profile of six transcripts.**

**Supplementary Figure 6. Chromatin state and MECP2 occupancy at DMRs.**

**A.** Heatmap for MECP2 ChIP and MNase tag intensity at 311 DMRs. **B.** MECP2 tag intensity at 64 overlapping DMRs. **C.** MNase tag intensity at 64 overlapping DMRs. **D.** MECP2 ChIP and MNase tag intensity distribution at DMRs. Plot is extended 5kb up and 5kb downstream at x axis.

**Supplementary Figure 7. *Gnas* isoform specific methylation difference.**

**A.** *Gnas* transcript variants. **B.** *Gnas6* Δmethylation distribution from two comparisons. **C.** *Gnas9* Δmethylation distribution from two comparisons.
