# Supplementary material for: Prenatal immune activation alters the adult neural epigenome but can be partly stabilised by a n-3 polyunsaturated fatty acid diet
Source: Transl Psychiatry. 2018 Jul 2;8:125. doi: 10.1038/s41398-018-0167-x (PMC6028639; doi:10.1038/s41398-018-0167-x)
Supplement: Supplementary file 8 — Supplementary Figure 1 [file 41398_2018_167_MOESM8_ESM.pptx]

## Slide 1
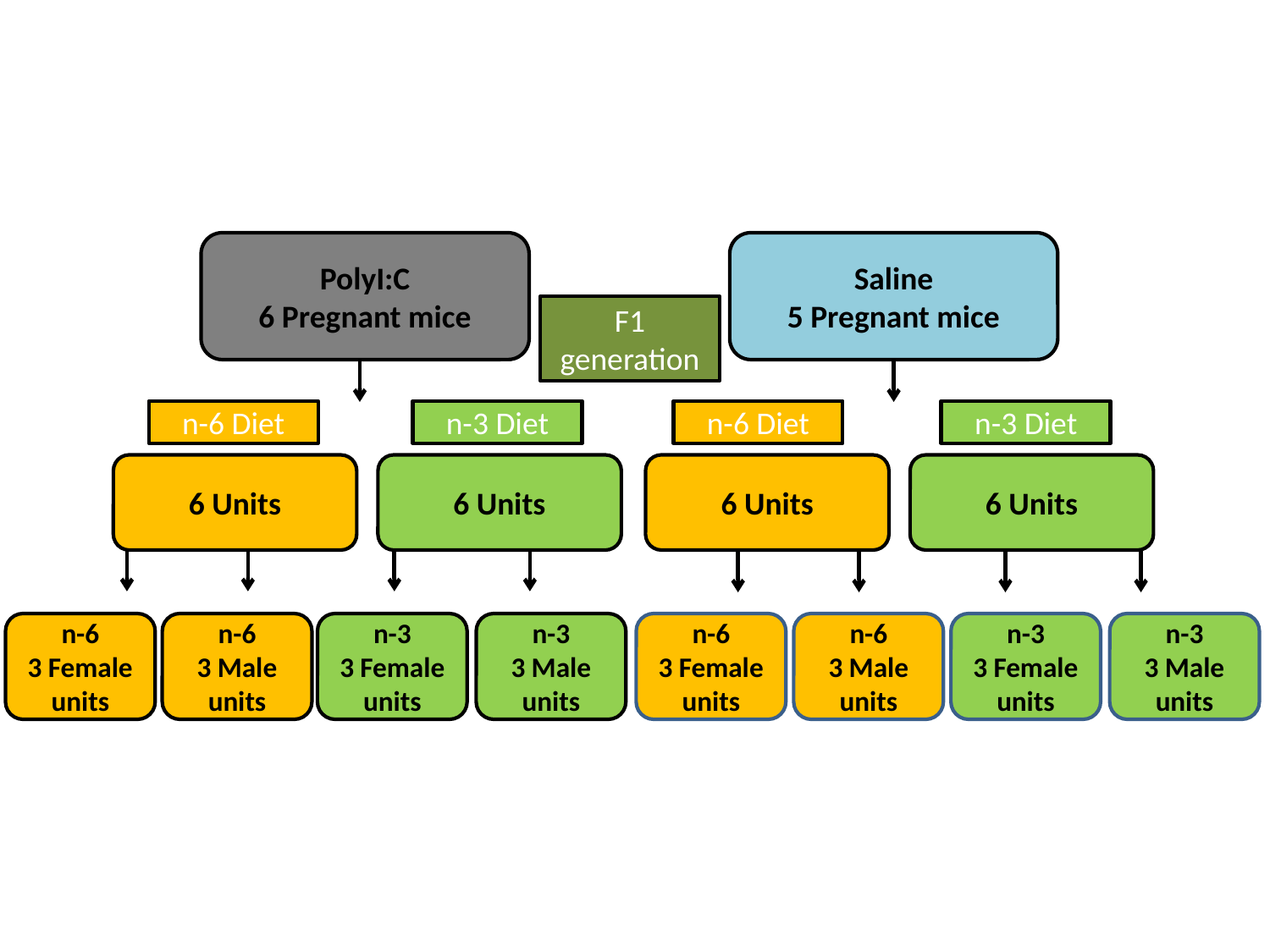

PolyI:C
6 Pregnant mice
Saline
5 Pregnant mice
F1 generation
 n-6 Diet
 n-3 Diet
 n-6 Diet
 n-3 Diet
6 Units
6 Units
6 Units
6 Units
n-6
3 Female units
n-6
3 Male
units
n-3
3 Female
units
n-3
3 Male
units
n-6
3 Female
units
n-6
3 Male
units
n-3
3 Female
units
n-3
3 Male
units
