# Supplementary figures and images for: Prenatal immune activation alters the adult neural epigenome but can be partly stabilised by a n-3 polyunsaturated fatty acid diet
Source: Transl Psychiatry. 2018 Jul 2;8:125. doi: 10.1038/s41398-018-0167-x (PMC6028639; doi:10.1038/s41398-018-0167-x)

## Slide 1
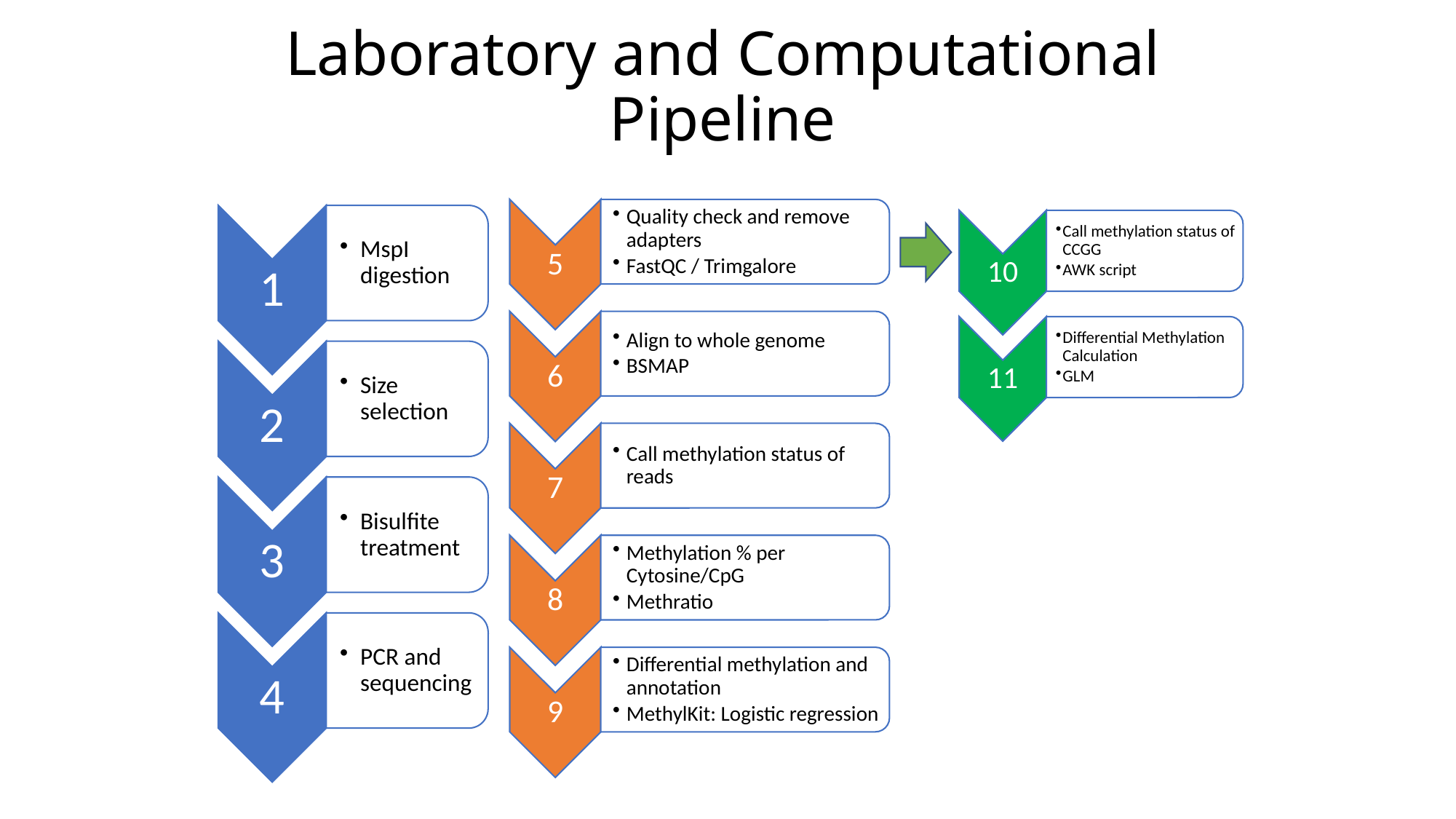

# Laboratory and Computational Pipeline

Supplement: Supplementary file 9 — Supplementary Figure 2 [file 41398_2018_167_MOESM9_ESM.pptx]

## Slide 1
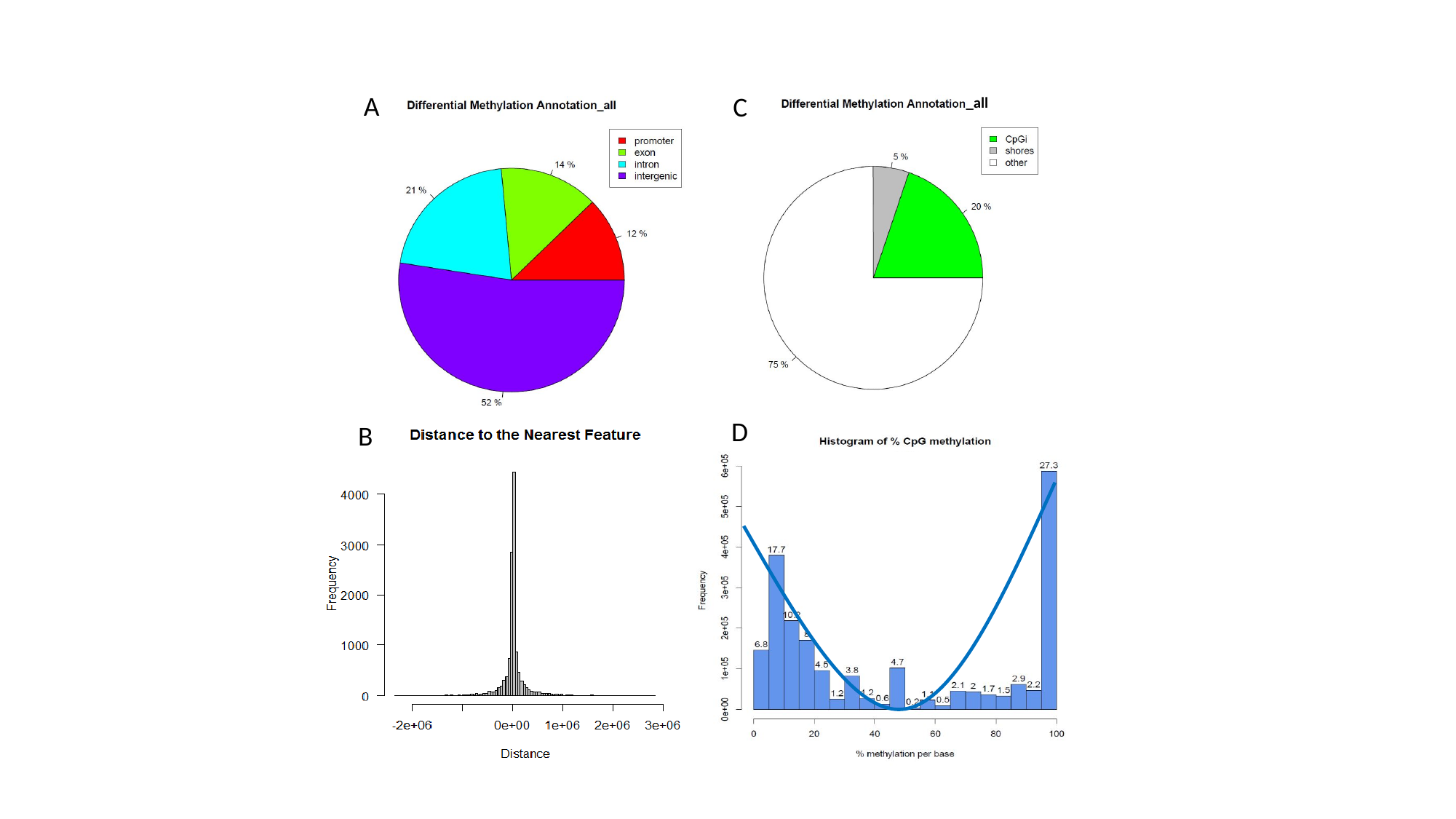

A
C
_all
D
B

Supplement: Supplementary file 10 — Supplementary Figure 3 [file 41398_2018_167_MOESM10_ESM.pptx]

## Slide 1
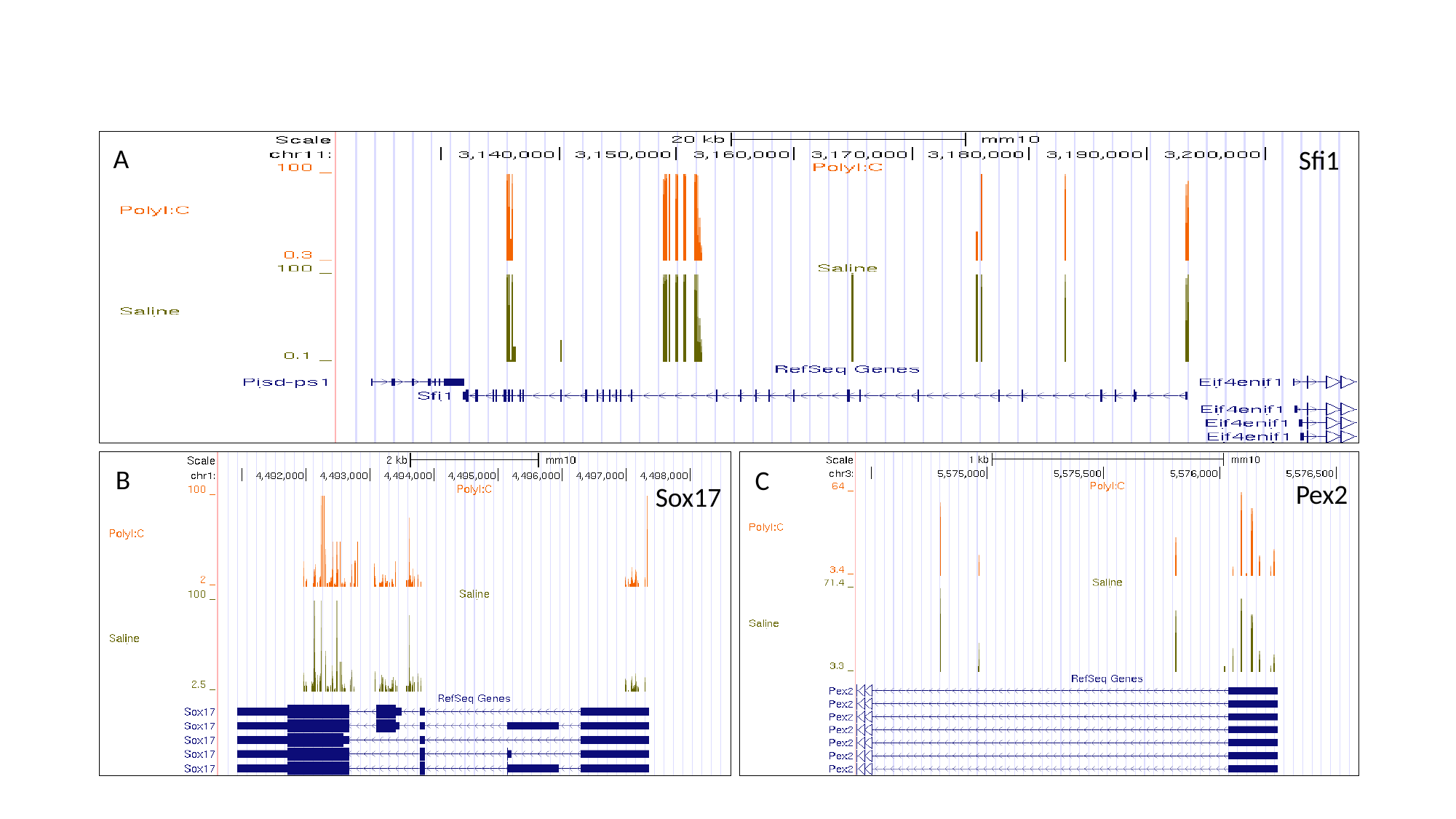

A
Sfi1
Sox17
B
C
Pex2
Sox17

Supplement: Supplementary file 11 — Supplementary Figure 4 [file 41398_2018_167_MOESM11_ESM.pptx]
