# Supplementary material for: Prenatal immune activation alters the adult neural epigenome but can be partly stabilised by a n-3 polyunsaturated fatty acid diet
Source: Transl Psychiatry. 2018 Jul 2;8:125. doi: 10.1038/s41398-018-0167-x (PMC6028639; doi:10.1038/s41398-018-0167-x)
Supplement: Supplementary file 12 — Supplementary Figure 5 [file 41398_2018_167_MOESM12_ESM.pptx]

## Slide 1
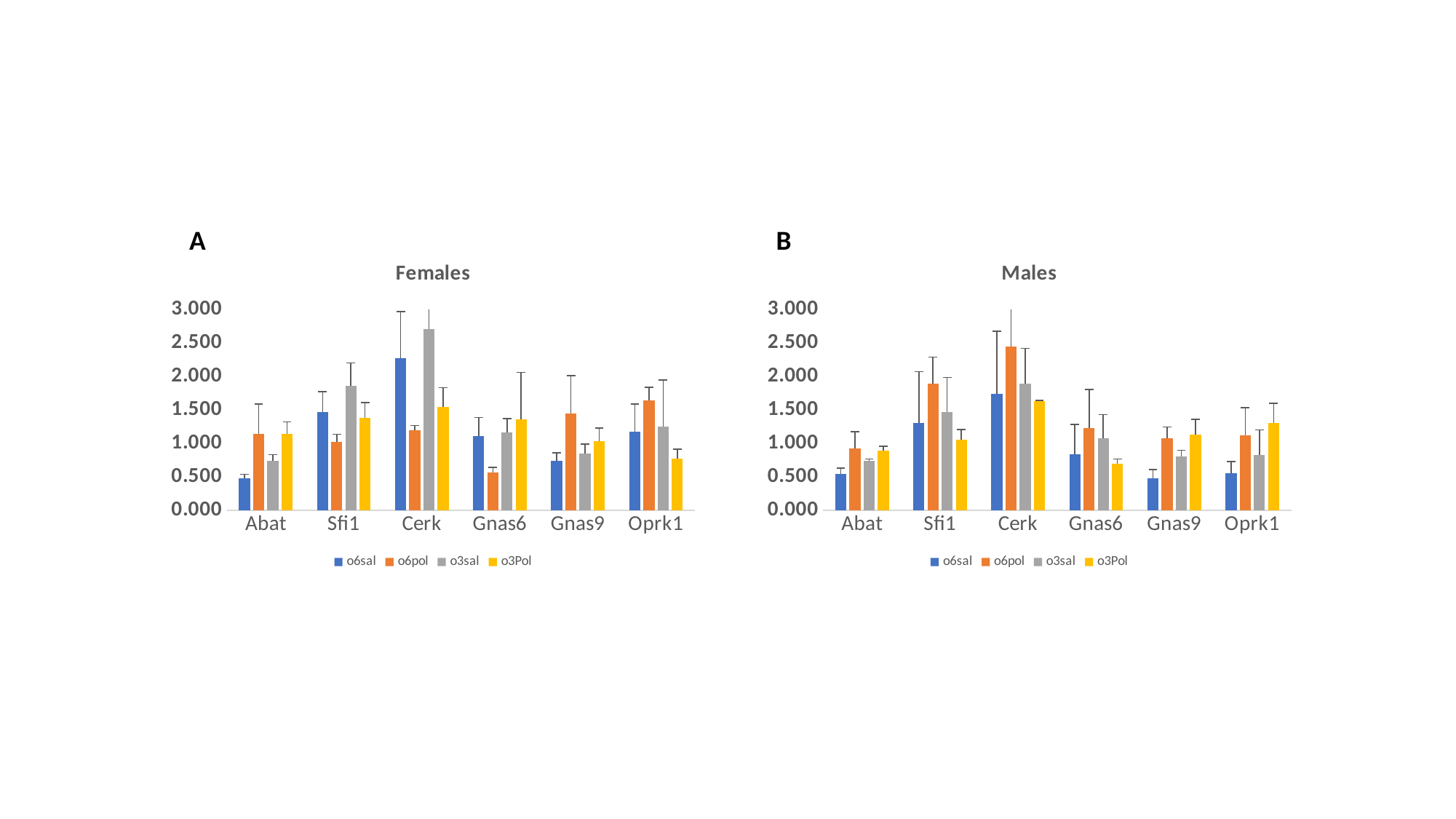

A
B
### Chart: Females
| Category | o6sal | o6pol | o3sal | o3Pol |
|---|---|---|---|---|
| Abat | 0.47995717451074643 | 1.1395953389378493 | 0.7432135311364295 | 1.1449814359794224 |
| Sfi1 | 1.4740340962051965 | 1.0236667203723198 | 1.8678420339611155 | 1.3839778498792625 |
| Cerk | 2.279081291362587 | 1.197044815773669 | 2.710942069115461 | 1.549354008393097 |
| Gnas6 | 1.1121866602210635 | 0.5684076371891295 | 1.1693147701475641 | 1.3566404313834728 |
| Gnas9 | 0.7468575399849765 | 1.4531020287249223 | 0.8501222459754002 | 1.0322343372636142 |
| Oprk1 | 1.1765632453490222 | 1.6473662281685906 | 1.257828945215041 | 0.7772282545282009 |
### Chart: Males
| Category | o6sal | o6pol | o3sal | o3Pol |
|---|---|---|---|---|
| Abat | 0.5507027500273596 | 0.9268032431900145 | 0.7442005537847324 | 0.8943644967259475 |
| Sfi1 | 1.3091427856729672 | 1.8921089214314748 | 1.475720939716883 | 1.0516610795547428 |
| Cerk | 1.7415896690274064 | 2.4494206728667782 | 1.8982246155167048 | 1.6385240834978845 |
| Gnas6 | 0.8348578084204932 | 1.228943299672294 | 1.0766229863592949 | 0.7025457437912803 |
| Gnas9 | 0.4858824632990948 | 1.0800933471814396 | 0.8063752320851713 | 1.132276023494267 |
| Oprk1 | 0.5568781952066039 | 1.1247779846955082 | 0.8248588707513657 | 1.3072315014590274 |
