# Supplementary material for: Prenatal immune activation alters the adult neural epigenome but can be partly stabilised by a n-3 polyunsaturated fatty acid diet
Source: Transl Psychiatry. 2018 Jul 2;8:125. doi: 10.1038/s41398-018-0167-x (PMC6028639; doi:10.1038/s41398-018-0167-x)
Supplement: Supplementary file 13 — Supplementary Figure 6 [file 41398_2018_167_MOESM13_ESM.pptx]

## Slide 1
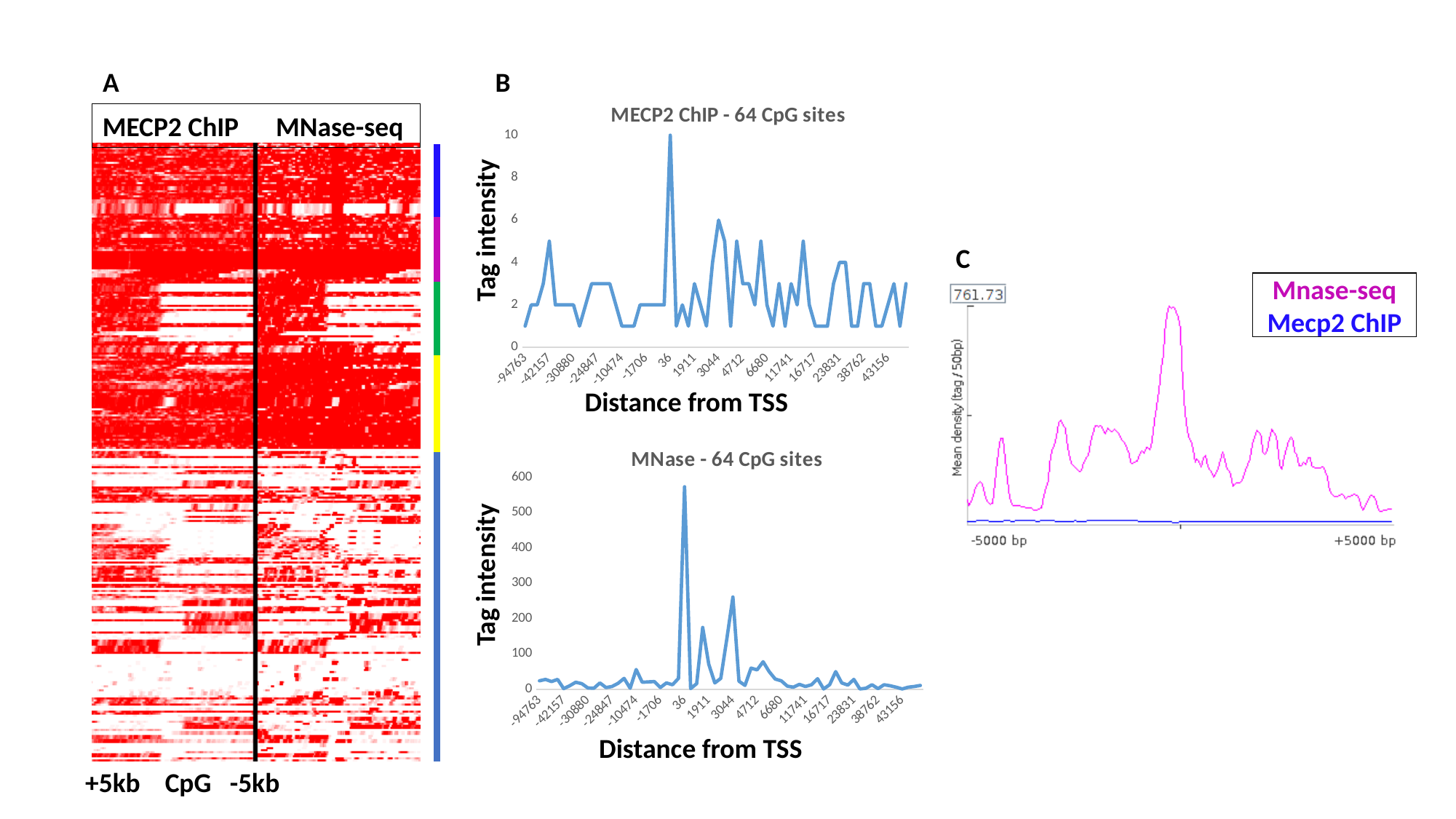

A
B
### Chart: MECP2 ChIP - 64 CpG sites
| Category | ChIP |
|---|---|
| -94763 | 1.0 |
| -94740 | 2.0 |
| -91317 | 2.0 |
| -48170 | 3.0 |
| -42157 | 5.0 |
| -31953 | 2.0 |
| -31044 | 2.0 |
| -30884 | 2.0 |
| -30880 | 2.0 |
| -30738 | 1.0 |
| -28944 | 2.0 |
| -26298 | 3.0 |
| -24847 | 3.0 |
| -21571 | 3.0 |
| -15284 | 3.0 |
| -11394 | 2.0 |
| -10474 | 1.0 |
| -9086 | 1.0 |
| -8990 | 1.0 |
| -2460 | 2.0 |
| -1706 | 2.0 |
| -990 | 2.0 |
| -473 | 2.0 |
| -11 | 2.0 |
| 36 | 10.0 |
| 94 | 1.0 |
| 540 | 2.0 |
| 1854 | 1.0 |
| 1911 | 3.0 |
| 2041 | 2.0 |
| 2673 | 1.0 |
| 3032 | 4.0 |
| 3044 | 6.0 |
| 3085 | 5.0 |
| 3905 | 1.0 |
| 3955 | 5.0 |
| 4712 | 3.0 |
| 4740 | 3.0 |
| 6247 | 2.0 |
| 6289 | 5.0 |
| 6680 | 2.0 |
| 9202 | 1.0 |
| 9477 | 3.0 |
| 11323 | 1.0 |
| 11741 | 3.0 |
| 14998 | 2.0 |
| 16105 | 5.0 |
| 16454 | 2.0 |
| 16717 | 1.0 |
| 17758 | 1.0 |
| 18207 | 1.0 |
| 20423 | 3.0 |
| 23831 | 4.0 |
| 27787 | 4.0 |
| 33548 | 1.0 |
| 36720 | 1.0 |
| 38762 | 3.0 |
| 40280 | 3.0 |
| 40869 | 1.0 |
| 41894 | 1.0 |
| 43156 | 2.0 |
| 45380 | 3.0 |
| 52535 | 1.0 |
| 72936 | 3.0 |Distance from TSS
Tag intensity
MECP2 ChIP MNase-seq
+5kb CpG -5kb
C
Mnase-seq
Mecp2 ChIP
### Chart: MNase - 64 CpG sites
| Category | MNase |
|---|---|
| -94763 | 24.0 |
| -94740 | 28.0 |
| -91317 | 22.0 |
| -48170 | 28.0 |
| -42157 | 2.0 |
| -31953 | 10.0 |
| -31044 | 20.0 |
| -30884 | 16.0 |
| -30880 | 4.0 |
| -30738 | 3.0 |
| -28944 | 18.0 |
| -26298 | 5.0 |
| -24847 | 8.0 |
| -21571 | 17.0 |
| -15284 | 31.0 |
| -11394 | 3.0 |
| -10474 | 56.0 |
| -9086 | 20.0 |
| -8990 | 21.0 |
| -2460 | 22.0 |
| -1706 | 5.0 |
| -990 | 18.0 |
| -473 | 13.0 |
| -11 | 31.0 |
| 36 | 573.0 |
| 94 | 2.0 |
| 540 | 16.0 |
| 1854 | 175.0 |
| 1911 | 71.0 |
| 2041 | 18.0 |
| 2673 | 31.0 |
| 3032 | 143.0 |
| 3044 | 261.0 |
| 3085 | 23.0 |
| 3905 | 11.0 |
| 3955 | 60.0 |
| 4712 | 55.0 |
| 4740 | 78.0 |
| 6247 | 50.0 |
| 6289 | 29.0 |
| 6680 | 24.0 |
| 9202 | 9.0 |
| 9477 | 6.0 |
| 11323 | 14.0 |
| 11741 | 8.0 |
| 14998 | 13.0 |
| 16105 | 30.0 |
| 16454 | 1.0 |
| 16717 | 13.0 |
| 17758 | 50.0 |
| 18207 | 18.0 |
| 20423 | 12.0 |
| 23831 | 28.0 |
| 27787 | 1.0 |
| 33548 | 3.0 |
| 36720 | 13.0 |
| 38762 | 2.0 |
| 40280 | 13.0 |
| 40869 | 10.0 |
| 41894 | 6.0 |
| 43156 | 1.0 |
| 45380 | 6.0 |
| 52535 | 8.0 |
| 72936 | 11.0 |Distance from TSS
Tag intensity
