# Supplementary material for: Prenatal immune activation alters the adult neural epigenome but can be partly stabilised by a n-3 polyunsaturated fatty acid diet
Source: Transl Psychiatry. 2018 Jul 2;8:125. doi: 10.1038/s41398-018-0167-x (PMC6028639; doi:10.1038/s41398-018-0167-x)
Supplement: Supplementary file 14 — Supplementary Figure 7 [file 41398_2018_167_MOESM14_ESM.pptx]

## Slide 1
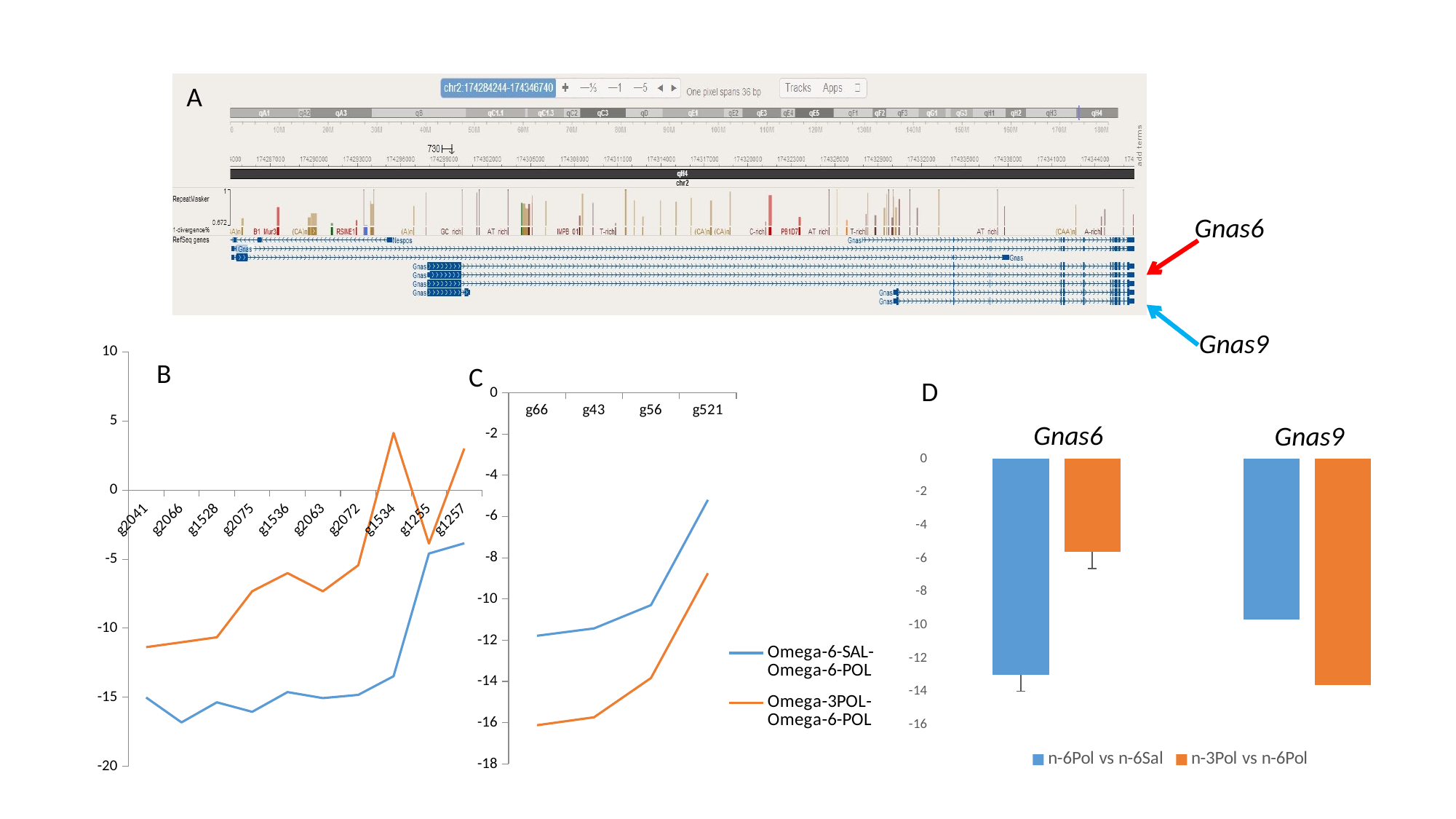

A
Gnas6
Gnas9
### Chart
| Category | Omega-6-SAL-Omega-6-POL | Omega-3POL-Omega-6-POL |
|---|---|---|
| g2041 | -15.01683023 | -11.37296867 |
| g2066 | -16.81781433 | -11.01709678 |
| g1528 | -15.36345988 | -10.65500336 |
| g2075 | -16.05153081 | -7.322133386999999 |
| g1536 | -14.62467645 | -6.013513513999999 |
| g2063 | -15.05906033 | -7.330567082 |
| g2072 | -14.82653169 | -5.447154471999999 |
| g1534 | -13.47605695 | 4.135338345999999 |
| g1255 | -4.589272189 | -3.870187462 |
| g1257 | -3.849066996999999 | 3.007518797 |B
C
D
### Chart
| Category | Omega-6-SAL-Omega-6-POL | Omega-3POL-Omega-6-POL |
|---|---|---|
| g66 | -11.78571429 | -16.12316325 |
| g43 | -11.42857143 | -15.73706286 |
| g56 | -10.29985373 | -13.83549615 |
| g521 | -5.194455140999998 | -8.751608752 |Gnas6
Gnas9
### Chart
| Category | n-6Pol vs n-6Sal | n-3Pol vs n-6Pol |
|---|---|---|
| Gnas6 | -12.9674299856 | -5.5885767584 |
| Gnas9 | -9.67714864775 | -13.611832752999998 |
